# Design and Analysis of Novel HEV Vaccine Variants and Evaluation of Two Selected Candidates in a Porcine Infection Model

**DOI:** 10.1111/liv.70246

**Published:** 2025-08-01

**Authors:** Isabella Hrabal, Elmira Aliabadi, Saskia Weber, George Liam Ssebyatika, Thomas Krey, Cora M. Holicki, Laura Schmid, Katja Dinkelborg, Charlotte Schröder, Christine Fast, Patrick Behrendt, Martin H. Groschup, Martin Eiden

**Affiliations:** ^1^ Institute for Novel and Emerging Infectious Diseases, Friedrich‐Loeffler‐Institut Greifswald Germany; ^2^ Institute for Experimental Virology, TWINCORE, Centre for Experimental and Clinical Infection Research Hannover Germany; ^3^ Helmholz Center for Infection Research GmbH Braunschweig Germany; ^4^ Institute of Diagnostic Virology, Friedrich‐Loeffler‐Institut Greifswald Germany; ^5^ Institute of Biochemistry University of Lübeck Lübeck Germany; ^6^ Erasmus MC, Department of Viroscience Rotterdam the Netherlands; ^7^ Institute of Molecular Virology and Cell Biology, Friedrich‐Loeffler‐Institut Greifswald Germany; ^8^ Department of Gastroenterology, Hepatology, Infectious Diseases and Endocrinology Hannover Medical School Hannover Germany; ^9^ German Centre for Infection Research Partner Site Braunschweig‐Hannover Braunschweig Germany; ^10^ Department of Experimental Animal Facilities and Biorisk Management Friedrich‐Loeffler‐Institut Greifswald Germany; ^11^ German Centre for Infection Research Partner Site Hamburg‐Lübeck‐Borstel‐Riems Greifswald Germany

**Keywords:** animal model, hepatitis E virus, in vitro characterisation, pig infection model, vaccine

## Abstract

**Background and Aims:**

Hepatitis E virus (HEV) poses a significant global health concern, with millions of annual infections and a notable impact on public health. Although HEV is the leading cause of acute viral hepatitis worldwide, there is a substantial lack of approved and licensed vaccines. In this study, we evaluated the efficacy of several protein‐ and DNA‐based vaccine candidates against HEV using a combined in vitro/in vivo workflow.

**Methods:**

Corresponding vaccine candidates were produced, biochemically analysed and characterised. The general immunogenicity of suitable vaccine candidates was initially evaluated in a rabbit model. Resulting antibodies were assessed for their reactivity and neutralising efficiency. Finally, the most effective candidates were tested in a pig infection model using a prime‐boost vaccination regimen.

**Results:**

Using this approach, we analysed a total of seven vaccine candidates and demonstrated that the two most promising candidates significantly reduced virus shedding in swine faecal samples after infection. However, no sterile immunity was achieved.

**Conclusions:**

This study conducted a comprehensive analysis to establish a rational approach for post‐vaccination immune responses in pigs. The insights gained from this research are expected to significantly contribute to the development and evaluation of future vaccine candidates for pig herds, ultimately reducing viral dissemination among pigs and preventing HEV transmission from pigs to humans. These findings hold important translational value, offering a foundation for both improving animal health and safeguarding public health.

**Summary**

- In this study, a comprehensive method was developed to select and evaluate new vaccine candidates against HEV‐3.
- These candidates underwent rigorous in vitro characterisation followed by thorough testing in two animal models.

**Abbreviations**
BSL: biosafety level
ddPCR: digital droplet PCR
DNA: desoxyribonucleic acid
dpv: days post vaccination

*E. coli*

:
*Escherichia coli*

ELISA: enzyme‐linked immunosorbent assay
FFU: focus forming unit
FLI: Friedrich‐Loeffler‐Institut
HEV: hepatitis E virus
HEV‐1–4: hepatitis E virus genotype 1–4
HZI: Helmholtz‐Centre for Infection Research
IgG: immunoglobulin G
MFI: mean fluorescence intensity
MHC: major histocompatibility complex
OD: optical density
ORF: open reading frame
PBS: phosphate buffered saline
qRT‐PCR: quantitative reverse transcription polymerase chain reaction
RNA: ribonucleic acid

## 1 Background and Aims

The hepatitis E virus (HEV) is a leading cause of acute viral hepatitis, particularly prevalent in developing countries. It is known to cause acute liver inflammation, ranging from a self‐limiting illness to a severe, potentially life‐threatening condition [1].

HEV belongs to the *Hepeviridae* family, which is divided into two subfamilies: *Orthohepevirinae* and *Parahepevirinae*. *Orthohepevirinae* encompasses four genera: *Avihepevirus, Chirohepevirus, Rocahepevirus* and *Paslahepevirus*. Within the genus *Paslahepevirus*, there are a total of eight genotypes, with genotypes 1–4 (HEV‐1‐4) being mainly associated with human infections [2]. HEV leads to frequent outbreaks in Africa and Asia, with considerable impact on public health, often connected to contaminated water sources [3].

The zoonotic genotypes HEV‐3 and HEV‐4 primarily infect pigs and wild boars as their main reservoir and are particularly widespread in industrialised countries [4, 5, 6]. In Germany, HEV‐3 has the highest prevalence, resulting in an estimated 417 000 seroconversions per year [7]. Transmission to humans primarily occurs through direct contact with the faeces of infected pigs or the consumption of undercooked pork products [8, 9, 10]. Although most HEV infections usually lead to self‐limiting diseases, chronic infections with HEV‐3 have been reported, especially in immunocompromised individuals, representing an additional burden for disease control [1].

The development of vaccines plays a crucial role in reducing the spread of the virus. In recent years, various vaccines against different HEV genotypes have been developed [11]. Notably, Hecolin (Xiamen Innovax Biotech Co., Xiamen, China), utilised in humans, has demonstrated a protective effect against HEV‐4 [12]. It has now been approved and licensed for humans in China for over a decade [12, 13] and has recently also been approved in Pakistan [14]. However, a recent publication has shown that the vaccine offers only partial protection against HEV‐3 in a pig model [15]. Further efforts are therefore necessary to develop an effective vaccine against all genotypes, including HEV‐3.

In this study, we focused on establishing a standardised workflow to evaluate seven new vaccine candidates. This included comprehensive in vitro analyses to assess the candidates, as well as an in vivo approach to determine their effectiveness in a pig infection model. We aimed for the development of a vaccine against HEV‐3 in pig herds to reduce viral dissemination between pigs, thereby mitigating the risk of infection in humans through a One Health approach.

## 2 Material and Methods

### 2.1 Animals

In total, seven rabbits were obtained from an internal Friedrich‐Loeffler‐Institut (FLI; Greifswald‐Riems, Germany) breeding programme and kept in the local husbandry. The competent authority of the Federal State of Mecklenburg‐Vorpommern, Germany, was notified based on national legislation (LALLF MV 7221.3‐2‐042/17).

Twenty‐two 11‐week‐old mast hybrid piglets were purchased from a commercial HEV‐negative herd from a local breeder (Landboden Glasin, Glasin, Germany). The experiment was approved by the State Office for Agriculture, Food Safety and Fishery in the Federal State of Mecklenburg‐Western Pomerania, Germany, based on national and European legislation, EURL 63/2010 for the protection of laboratory animals (LALLF M‐V 7221.3‐1‐010/22).

All possible steps were taken to improve animal wellbeing and to keep the number of animals in the experiment to the required minimum.

### 2.2 Vaccine Design

The design of the majority of the analysed vaccine candidates is based on a partial sequence of the capsid protein from a German HEV‐3 isolate (GenBank Acc. Number KP294371.1 [16]). This includes the two bacterial expressed open reading frame (ORF) 2 constructs p429 and p429‐ORF3, which encompass 429 amino acids of the central region of the ORF2, with or without fusion to ORF3, connected via a helix‐forming peptide linker. In addition, three DNA‐based vaccines, pVax1‐ub‐HEV‐SMP, pVax1‐ub‐HEV‐SMP‐ORF3 and pVax1‐ub‐HEV‐ORF3‐SMP, were prepared. They code for the 3 functional (S, M, P) capsid domains either solely or fused to ORF3 protein at the 3′ or 5′ end of the capsid sequence. The sequences were cloned into a modified pVAX1‐Ub universal fusion vector which enables expression of a 5′‐ubiquitin antigen fusion protein [17]. Corresponding sequences and details are compiled in the Sequences S1–S5.

In addition, non‐secreted and secreted forms of HEV‐3 P domain (pGS99/100, respectively) were cloned into a pMT vector and expressed in *Drosophila S2* cells as described previously [18]. The gene encodes the HEV‐3 P domain residues 456–660 (Kernow‐C1 clone p6, genotype 3, GenBank Acc. Number JQ679013). It was designed with and without a BiP signal sequence to represent the glycosylated (secreted: pGS100) and non‐glycosylated (non‐secreted: pGS99) form of the P domain. All vaccine variants are summarised in Table 1.

**TABLE 1:** Overview of generated vaccine candidates.

Name of vaccine	Type of vaccine	Inserted sequence	Aminoacid position ORF2	Aminoacid position ORF3
p429	Protein	Partial HEV capsid	178–606	—
p429‐ORF3	Protein	Partial HEV capsid fused with ORF3	178–606	30–122
pGS99	Protein	P‐domain of HEV capsid, non‐secreted, non‐glycosylated	456–660	—
pGS100	Protein	P‐domain of HEV capsid, secreted, glycosylated	456–660	—
pVax1‐ub‐HEV‐SMP	Plasmid	Ubiquitin‐antigen (capsid S/M/P domain)	129–605	—
pVax1‐ub‐HEV‐SMP‐ORF3	Plasmid	Ubiquitin‐antigen (capsid S/M/P domain, fused with ORF3)	129–605	30–122
pVax1‐ub‐HEV‐ORF3‐SMP	Plasmid	Ubiquitin‐antigen (ORF3 fused with capsid S/M/P domain)	129–605	30–122

### 2.3 Expression and Purification of Recombinant Proteins and Plasmids

Bacterial proteins and pVax1 constructs were expressed in *E. coli* , while eukaryotic proteins were expressed in *Drosophila S2* cells. All proteins and constructs were purified, diluted and stored at appropriate temperatures, with detailed cloning and purification methods provided in Method S1.

### 2.4 Rabbit Immunisation

The protein vaccines were administered by mixing 600 μL of the vaccine candidate [0.5 mg/mL] with 600 μL of Gerbu adjuvant MM (Biotechnik Gerbu, Heidelberg, Germany). This mixture was applied subcutaneously to one rabbit per vaccine candidate. Three successive subcutaneous boosts followed at intervals of 3 weeks. Blood samples were taken from the lateral saphenous vein to determine the development of antibody titers. The obtained serum samples were stored at −20°C for further analysis. The experiment design is shown in Figure 2A.

Plasmid vaccine pVax1‐ub‐HEV‐SMP was initially injected using the needle‐free PharmaJet injection system Stratis IM/SC (Colorado, USA) for all plasmid‐based vaccines. Using this system, 800 μL of plasmids was administered with the PharmaJet device into the semitendinosus muscle followed by 250 μL of the Gerbu adjuvant MM at the same application site while releasing the adjuvant when the needle was withdrawn. The vaccination cycle of 3 weeks continued until a plateau of serum antibodies was observed.

### 2.5 ELISA (Enzyme‐Linked Immunosorbent Assay)

An indirect antigen ELISA was performed according to standard protocol [19] using recombinant HEV antigens p239, p429 or 2xORF3 for coating (Sequences S1 and S6). A detailed description can be found in the Method S2. Additionally, a commercial ELISA (ID Screen Hepatitis E Indirect Multi‐species, ID.vet Innovative Diagnostics, Grabels, France) was used following the ID Screen manual.

### 2.6 Neutralisation Assay

Naked and pseudo‐enveloped HEV‐3 viral strain Kernow C1p6 G1634R was generated following the previously described method [20]. The analysis of serum samples as well as the counting of focus forming units (FFU) was performed as described in a recent publication [15]. Additional details can be found in the Method S3.

### 2.7 Immunofluorescent Staining

Human hepatoma HepG2/C3A cells were transfected with two HEV‐3 constructs (‘Kernow‐C1 p6 clone’ and ‘HEV83‐2‐27‐clone’) and treated with rabbit sera overnight, followed by staining and Mean Fluorescence Intensity (MFI) calculation as detailed in Method S6 and S7.

### 2.8 Inoculum

A 25% (w/v) liver inoculum of a HEV positive liver sample in phosphate buffered saline (PBS) was obtained from an experimentally HEV‐3 infected wild boar [16] (GenBank Acc. Number KP294371.1). The liver was homogenised in PBS using the TissueLyser II (Qiagen, Hilden, Germany), centrifuged at 7459×*g* for 5 min, pooled and filtered twice through syringe filters (0.22 μL Millex‐GP 33 mm filter unit, Carrigtwohill, Ireland). The obtained infectious homogenates were aliquoted into 2 mL portions and stored at −80°C. The corresponding inocula were thawed overnight at 4°C and brought to room temperature shortly before infection.

### 2.9 Molecular Analysis

RNA was extracted from various matrices using the NucleoVet Mag kit (Macherey Nagel, Düren, Germany) and KingFisher Flex robot (ThermoScientific, Darmstadt, Germany), then quantified by qRT‐PCR targeting a conserved ORF2/3 region [16], with β‐actin as a control. Standards for quantification were prepared using digital droplet PCR (ddPCR). A detailed description is found in Method S8.

### 2.10 Experimental Design of the Pig Vaccination Study

The experiment was performed under biosafety level (BSL)‐2 conditions in the corresponding animal facilities at the FLI, Germany. Twenty‐two 11‐week‐old HEV‐negative male and female mast hybrid pigs were divided into five experimental groups: Uninfected control group (*n* = 2), infected adjuvant control group (*n* = 2), infection control group (*n* = 6) and two vaccine groups (*n* = 6 each) (Table 2). The pigs were housed in separate stable units, each with 2–3 piglets of the same experimental group and gender. Vaccine groups received 300 μg of vaccine protein, mixed 1:1 (v/v) with Gerbu adjuvant F (Biotechnik Gerbu, Heidelberg, Germany). The experimental design is shown in Figure 2A and followed a recently established protocol [15]. Further details adhering to the ARRIVE guidelines including information on the pigs, health evaluation, termination criteria, acclimation period, vaccination regimen, sampling, euthanasia and necropsy are provided in Method S9 and Data S1.

**TABLE 2:** Overview of the pig vaccination trial.

Group	Number of animals	Gender	Administered vaccine	Age at vaccination	Infection	Age at infection	Animal identification (Eartag number)
Nontreated control	2	2× male	—	—	No	—	81 96
Adjuvant control	2	2× female	Dialysis buffer + Gerbu F adjuvant	Vaccination 1: 12 weeks Vaccination 2: 16 weeks	Yes	20 weeks	85 86
Infection control	6	3× female 3× male	—	—	Yes	20 weeks	15 17w 93 6 8 13
Vaccine 1 (p429)	6	3× female 3× male	P429 + Gerbu F adjuvant	Vaccination 1: 12 weeks Vaccination 2: 16 weeks	Yes	20 weeks	12b 14 20 7 16 17b
Vaccine 2 (p429‐ORF3)	6	6× male	P429‐ORF3 + Gerbu F adjuvant	Vaccination 1: 12 weeks Vaccination 2: 16 weeks	Yes	20 weeks	82 94 97 91 95 98

### 2.11 Statistical Evaluation

The statistical analysis was performed using R statistical software (R Core Team [2023]. _R: A Language and Environment for Statistical Computing_. R Foundation for Statistical Computing, Vienna, Austria) and GraphPad Prism (GraphPad Prism version 10.0.0 for Windows, GraphPad Software, Boston, Massachusetts, USA). Group comparisons were conducted using both the Wilcoxon Rank Sum Test and the Welch two‐sample t‐test (Method S10).

## 3 Results

### 3.1 Rabbits Immunisation Elicits Neutralisation IgG With High Avidity Against HEV Genotype 3

Following the immunisation of rabbits with seven vaccine candidates, serum samples were tested for HEV‐capsid‐directed antibodies using ELISA, with partial HEV capsid proteins p429 and p239 as the coating antigens. Additionally, ORF3‐directed antibodies were assessed using ELISA with 2xORF3 as the coating antigen. High optical densities (OD) in the ELISA were indicative of elevated levels of HEV‐directed antibodies, with an upper detection limit of an OD of 4. All vaccinations were administered subcutaneously. However, four subcutaneous vaccinations with the DNA‐based vaccine candidate pVax1‐ub HEV‐SMP was unsuccessful in producing detectable antibody formation; consequently, the administration route for DNA‐based vaccines was changed to intramuscular injection.

**FIGURE 1:**
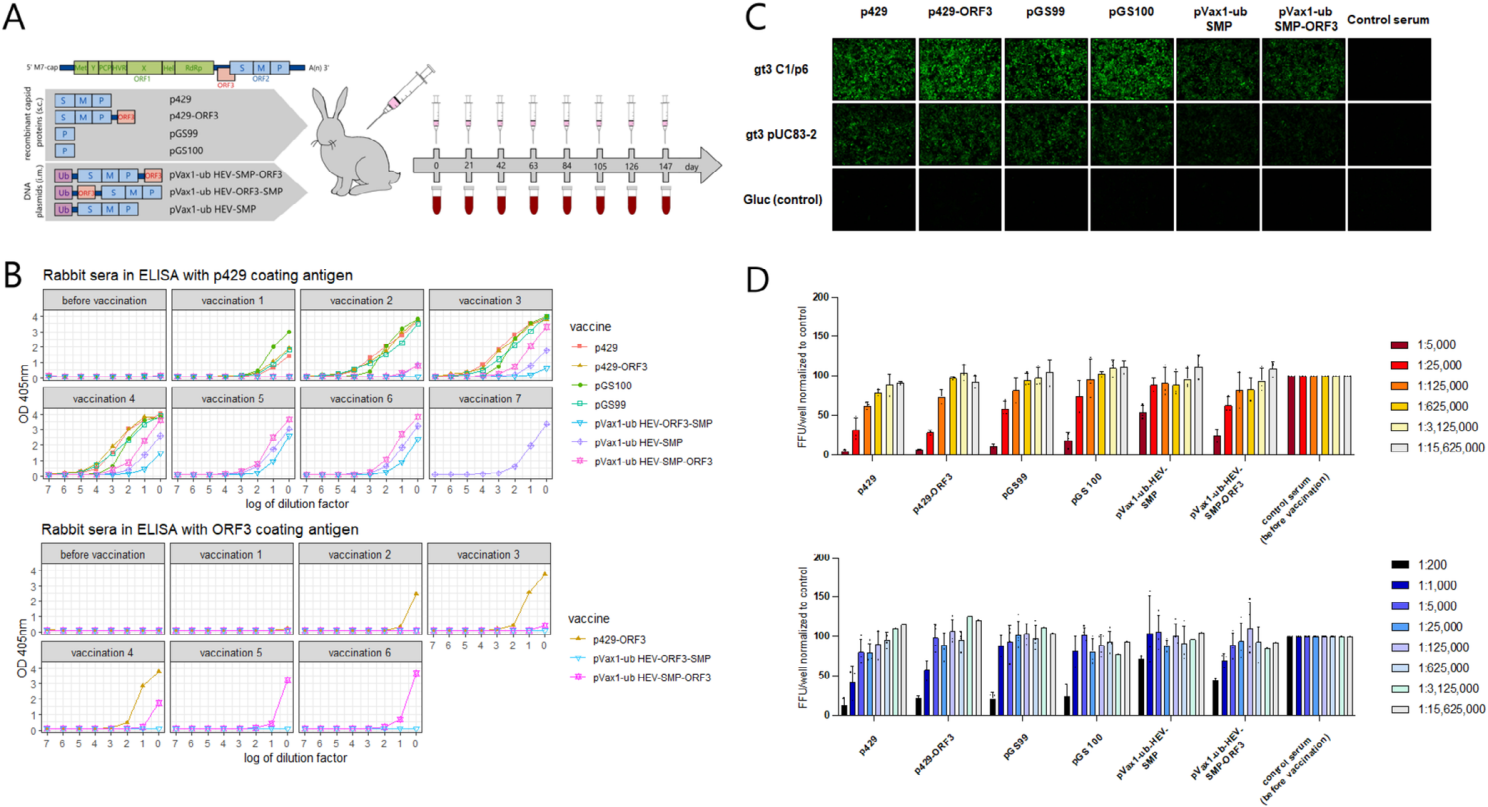
Immunisation of rabbits resulted in the generation of IgG antibodies exhibiting strong binding and neutralisation capabilities against HEV‐3. (A) Schematic representation of the applied prime boost strategy for the generation of polyclonal antibodies against the vaccine candidates. The vaccines were administered at 3‐week intervals, with blood collection prior to each vaccination. (B) ELISA with p429‐coating and 2xORF3‐coating. Serum samples were initially diluted 1:25 for the ELISA assays and further 10‐fold serially diluted (1 = 1:250, 2 = 1:2500, 3 = 1:25 000, 4 = 1:250.000, 5 = 1:2 500 000, 5 = 1:25 000 000, 6 = 1:250 000 000, 7 = 1:2 500 000 000). For the vaccine candidate pVax1‐ub HEV‐SMP, only intramuscular vaccinations were included in this figure. Immunisation of rabbits was stopped when a plateau was reached, indicated by the same course of the curve between immunizations. (C) Immunofluorescence staining of transfected hepatoma (HepG2) cells using rabbit sera targeting various sub‐genotypes of HEV‐3. (D) Neutralisation activity of rabbit sera from the latest serum sample against naked form (orange graph) and pseudo‐enveloped form (blue graph) of HEV‐3 viral strain Kernow C1p6 G1634R. Focus forming units (FFU) were quantified following incubation of the virus with sequential sera obtained from the immunised rabbits.

Immunisation (Figure 1A) resulted in an increase in p429‐directed antibodies across all seven vaccine candidates, as evidenced by higher optical density readings. However, the immunogenicity varied among the individual vaccines. Protein‐based vaccines induced capsid‐directed antibody production after a single vaccination, while DNA‐based vaccines showed increased antibody levels only after the second vaccination, with a slower rise of the OD compared to the protein vaccines. An antibody plateau in p429‐ELISA was reached after four subcutaneous vaccinations for all protein vaccines, six intramuscular vaccinations for pVax1‐ub HEV‐SMP‐ORF3 and pVax1‐ub HEV‐ORF3‐SMP, and seven intramuscular vaccinations for pVax1‐ub HEV‐SMP (Figure 1B), in addition to the four previous ineffective subcutaneous applications with this vaccine candidate (Figure S1A). The highest antibody titers were induced by the vaccines p429, p429‐ORF3 and pGS99, in which antibodies could still be detected at the highest serum dilution of 1:2 500 000. Overall, ODs between three and four were achieved in ELISA with p429 coating for all vaccine candidates except pVax1‐ub HEV‐ORF3‐SMP, which also did not lead to an increase in anti‐ORF3 antibodies (Figure 1B). These results were confirmed with a similar ELISA using the p239 partial capsid protein as the coating antigen (Figure S1B).

The binding specificity of rabbit antibodies to HEV‐3 capsid proteins was confirmed by western blotting against bacterially expressed capsid proteins p239, p429 and 2xORF3 (Method S5, Figures S2B and S3).

To assess whether the antibodies developed in the immunised rabbits could bind to HEV‐3 subtypes in a more authentic capsid conformation, we performed immunofluorescence staining of HEV‐transfected hepatoma (HepG2) cells. The DNA‐based vaccine candidate pVax1‐ub HEV‐ORF3‐SMP was not included in this assay, since it performed poorly compared to the other vaccine candidates in previous analyses. The results demonstrated that antibodies in serum samples from immunised rabbits with all protein‐based and DNA‐based vaccine candidates could bind to both HEV Kernow C1p6 G1634R and HEV pUC83‐2 (Figure 1C). Moreover, the mean fluorescent intensity (MFI) was stronger for the samples obtained from rabbits immunised with the protein‐based vaccine, compared to the DNA‐based vaccine, which is consistent with the ELISA results (Figure S4).

We performed a comprehensive analysis to assess the neutralisation capacity of the sera from the rabbits immunised with different vaccine candidates against HEV after the last immunisation (Figure 1D). In our assay, all vaccinated rabbits developed neutralising activity against HEV‐3. Notably, rabbits inoculated with the protein‐based vaccines p429 and p429‐ORF3 appeared to exhibit enhanced neutralisation efficacy compared to the other vaccine candidates, as observed in the graphical data. The dilution of the serum at which the antibodies were able to neutralise 50% of the naked and pseudo‐enveloped viral infection (ID‐50) is summarised in Figure S5.

To evaluate the avidity of the rabbit sera antibodies towards HEV, we utilised a urea‐based avidity ELISA, employing different HEV epitopes as coating antigens including pGS99, p429, or p239 (Method S4). The titration curves of the rabbit serum samples from the various animals immunised with distinct vaccines are shown in Figure S6A. All rabbit sera showed strong binding towards pGS99 in the ELISA without urea incubation. Notably, the signal intensity did not decrease after incubation of the sera‐antigen mixture with an 8 M urea solution, pointing to a high avidity of the antibody–antigen complex. The avidity indices are displayed in Figure S6B, revealing that the p429 vaccine induced antibodies with the highest avidity. Similar results were determined for the binding of rabbit sera to p239 and p429 antigens. A summary of avidity indices in ELISA using p239 and p429 is provided in the table in Figure S6B.

### 3.2 Anti‐HEV Antibody Increase in Pigs After Initial Vaccination With p429 and p429‐ORF3, Followed by Decline Post‐Booster and Infection

**FIGURE 2:**
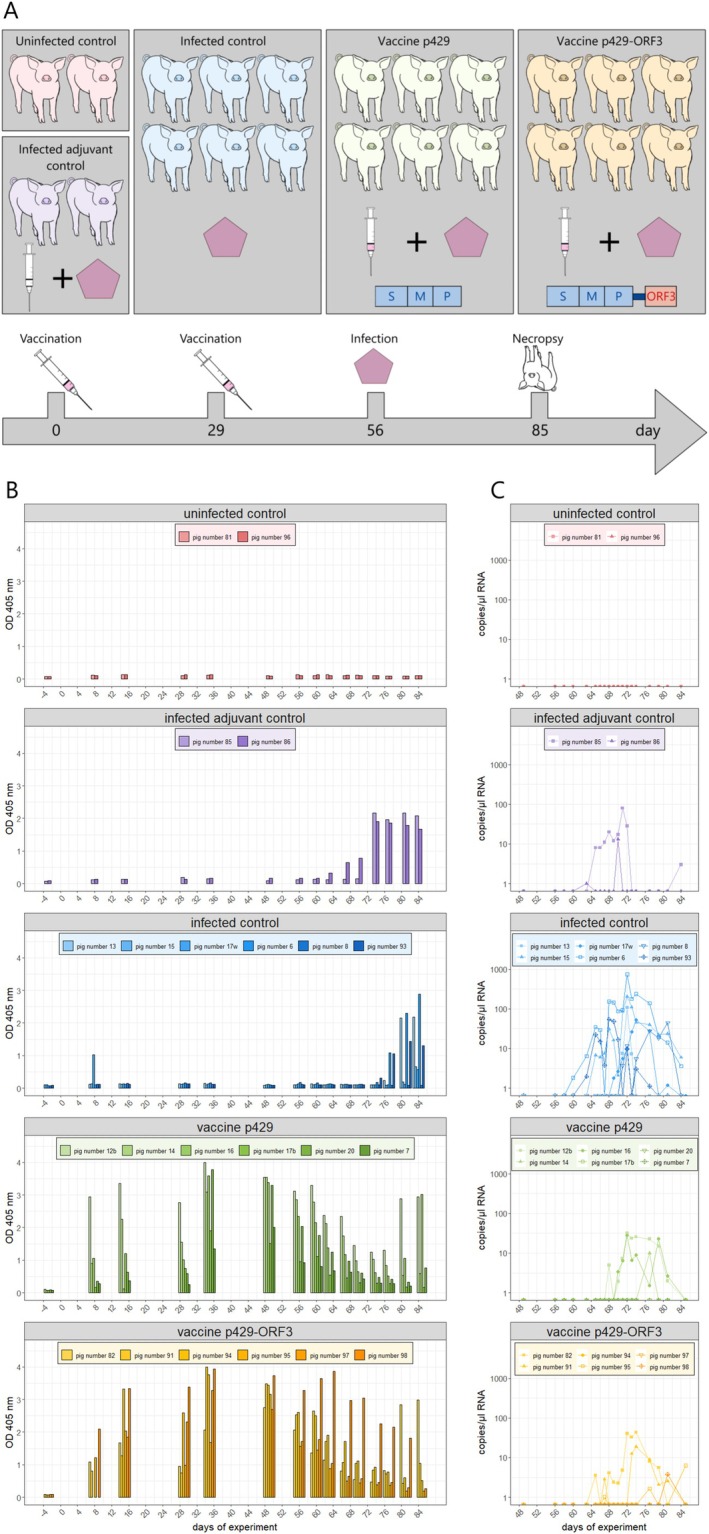
Immunisation of pigs with p429 and p429‐ORF3. (A) Experimental workflow of the pig vaccination study. Pigs were divided into 5 groups: Pigs vaccinated with vaccine candidate p429 (‘Vaccine p429’, *n* = 6), pigs vaccinated with vaccine candidate p429‐ORF3 (‘Vaccine p429‐ORF3’, *n* = 6), pigs infected without previous vaccination (‘infection control’, *n* = 6), pigs vaccinated only with adjuvant and subsequent infection (‘Infected adjuvant control’, *n* = 2) and a nontreated control to monitor viral carry‐over (‘uninfected control’, *n* = 2). (B) ELISA of pig sera with p239‐coating during vaccination study, sorted by group. (C) Detection of viral RNA in faecal samples of pigs after infection, sorted by group.

All protein vaccines elicited a similarly rapid increase in antibody response in rabbits compared to the DNA‐based vaccines and showed comparable performance in p239‐ELISA, Western blot and immunofluorescent staining. Overall, these results emphasise the highest potential of p429 and p429‐ORF3 to elicit protective immunity against HEV‐3: they were thus selected as the most promising vaccine candidates for further investigation in a pig infection model. They were freshly expressed and dialysed in carbonate bicarbonate buffer pH 10.3 (Figure S2A). The vaccination regimen in the pigs involved two intramuscular administrations, 28 days apart, with an infection 4 weeks after the second vaccination (Figure 2A).

Antibody titers against the vaccine candidates were evaluated using the p239 ELISA (Figure 2B) and the commercial ID Screen Hepatitis E Indirect Multi‐species ELISA (Figure S7A) between vaccinations and after infection. Both ELISAs showed that the homologous prime‐boost vaccination strategy initially led to an increase in antibody titers in all six vaccinated pigs across both vaccine groups. However, following this initial increase, antibody titers began to decrease after the booster vaccination and continued to decline after the infection on Day 56. Two pigs in the p429 vaccine group (pig 12b and pig 16), as well as one pig in the p429‐ORF3 vaccine group (pig 82), exhibited a detectable rise in antibody titers at the end of the observation period. Antibody titers also increased in the infection control group as well as in the adjuvant control group at the end of the observation period, with the rise occurring earlier in the adjuvant control group.

In addition, an ELISA was performed with 2xORF3 as the coating antigen (Figure S7B). None of the pigs in the infection control group exhibited detectable antibodies against 2xORF3. In contrast, two pigs from the P429‐ORF3 vaccinated group (pig 95 and pig 98) showed a slight increase in ORF3 antibodies after vaccination, which subsequently decreased in the further course of the trial.

### 3.3 Immunisation With p429 and p429‐ORF3 Vaccines Induces Neutralising Antibodies in Pigs

**FIGURE 3:**
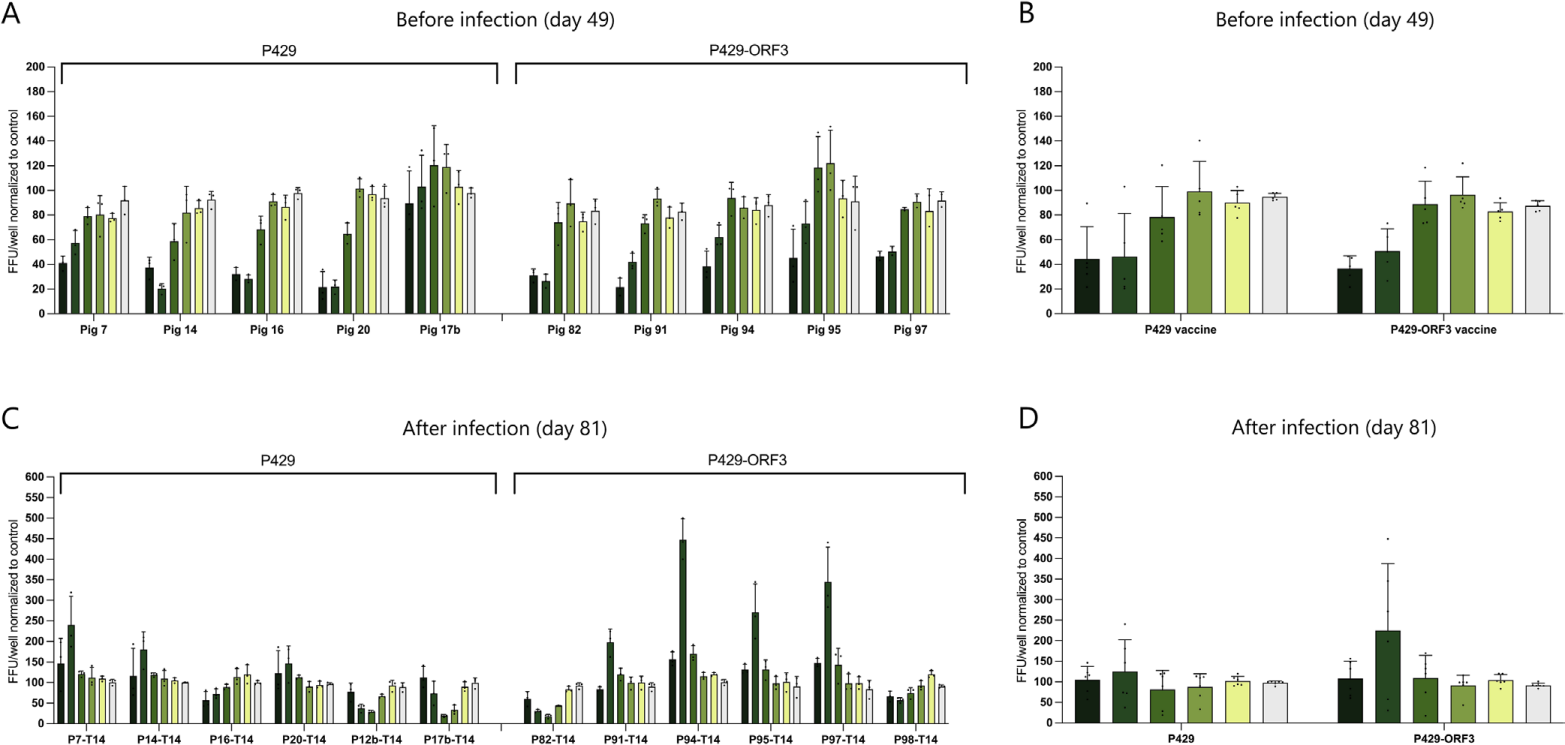
Neutralisation activity of pig sera against the naked form of HEV‐3 viral strain Kernow C1p6 G1634R on Day 49 post‐vaccination. Colours represent the dilution of serum samples, starting with 1:300 (dark green) in three‐fold serial dilutions to 1:72 000 (white). (A and B) and on Day 81 post‐vaccination (C and D). FFUs were quantified following the incubation of the virus with sequential sera from pigs immunised with the p429 or p429‐ORF3 vaccines. Results are normalised to the average neutralisation activity of two pigs from the adjuvant control group on the respective day. Each data point in the A and C graphs represents the mean of duplicate values from one experimental replicate. Each data point in the B and D graphs represents one pig.

To evaluate the humoral immune responses caused by the p429 and p429‐ORF3 vaccines in pigs, we assessed the neutralisation capacity of IgGs isolated from pig sera at 49 days post‐vaccination (dpv) before exposure to HEV‐3. The results indicate that IgGs from all vaccinated pigs, with the exception of pig 17b, were capable of neutralising the virus in a dose‐dependent manner. The data were normalised to the adjuvant control at 49 dpv to eliminate any nonspecific neutralisation caused by the adjuvants (Figure 3A). The neutralisation capacity of IgGs from each pig is combined in Figure 3B, where each dot represents one pig. The findings demonstrate that both vaccine candidates induced neutralising antibodies, with no apparent differences between the two vaccines, as observed in the graphical data.

Subsequently, we investigated whether HEV antibodies present in the pig sera after the second vaccination and subsequent infection exhibited neutralisation activity. For this analysis, the data for the vaccine groups were normalised to the infected adjuvant control at 81 dpv to account for any nonspecific neutralisation attributed to antibodies induced by the infection. At this time point, no obvious differences were observed between the vaccine groups and the infected adjuvant control. This suggests that although the vaccines induced neutralising antibodies, these antibodies did not enhance the overall neutralisation capacity following infection (Figure 3C,D).

Additionally, the avidity of antibodies in pig serum samples after vaccination was determined using a urea‐based avidity ELISA. The titration curves obtained from the average of the avidity ELISA experiments per vaccine group are depicted in Figure S8, with individual titrations from each pig shown in Figure S9. The data indicate that antibodies in the pig sera from both vaccine groups were capable of binding to all HEV antigens, with the strongest signal observed for the p239 coating protein. However, the avidity indices were only half those observed in immunised rabbits, suggesting that the binding strength of vaccine‐induced antibodies was higher in rabbits than in pigs.

### 3.4 p429 and p429‐ORF3 Vaccination Reduces Viral Load Post‐Infection

Viral shedding in faeces and serum was monitored throughout the experiment using RT‐qPCR (Figure 2C). Three animals in the p429‐ORF3 vaccine group exhibited mild diarrhoea on Day 61 (Table S3, Data S1). Viral RNA in faecal samples was detectable in all pigs (*n* = 6) of the non‐vaccinated infection control group and all pigs (*n* = 2) from the adjuvant control group between Days 58 and 84. In contrast, only three out of six p429‐vaccinated pigs and four out of six p429‐ORF3‐vaccinated pigs displayed faecal virus shedding. Moreover, the onset of virus shedding was delayed 2–16 days compared to the non‐vaccinated infection control group. Viremia was detectable only on one day in one pig in the infection control group (pig number 6) and one pig in the p429‐ORF3 vaccine group (pig number 82, Figure S7C).

Examination of the viral load in the faeces showed a significant reduction of viral RNA in the vaccinated pigs of both vaccination groups compared to the unvaccinated control group in both tests (*p* < 0.01). Statistics of faecal samples remained significant after exclusion of pigs with no HEV‐derived RNA in faeces, serum or organs (pigs 7 and 20 from the p429 vaccine group and pig 97 from the p429‐ORF3 vaccine group). A summary of the *p*‐values of all statistical tests performed is presented in Table S2. Differences in faecal HEV RNA between infected adjuvant control and vaccinated pigs were also found (*t*‐test: p429‐vaccinated pigs *p* < 0.1, p429‐ORF3 vaccinated pigs *p* < 0.05; Wilcoxon test: p429‐vaccinated pigs *p* < 0.1). However, it should be noted that exact food intake and stool volume were not monitored during the observation period. Therefore, a dilution effect cannot be excluded.

After necropsy, the viral load of organ samples (brain, kidney, liver, cranial mesenteric lymph node, spleen, gallbladder wall and bile) was determined (Method S9, Table S1). HEV‐RNA was detected in the bile of three out of six animals in the infection control group, one (p429 vaccination group) and three pigs (p429‐ORF3 vaccination group) in the vaccinated groups. No viral RNA was detected in the bile of the infected adjuvant control group. However, one pig in this group showed a low viral load in the liver.

The Wilcoxon Rank Sum Test revealed significant differences in viral load in liver (*p* < 0.1) and bile (*p* < 0.05) between the infected adjuvant control and both vaccine groups.

## 4 Discussion

Although hepatitis E is a globally prevalent virus and one of the most common causes of acute hepatitis, there is currently no globally licensed vaccine on the market. Hecolin, the only registered vaccine, is available only in China and Pakistan with proven efficiency against HEV‐4 [12, 21]. Hecolin was evaluated in numerous studies, demonstrating efficacy against HEV‐1 and rabbit HEV strains [22, 23, 24]. However, data on its effectiveness against HEV‐3 are limited [14]. A recent publication demonstrated that Hecolin and a similar HEV‐3‐based vaccine (p239 Riems) showed only limited protection against HEV‐3 in pigs. Notably, both vaccines induced antibodies capable of neutralising HEV‐3 isolates in vitro [15, 25].

Therefore, there is a significant gap in the availability of an effective vaccine against HEV‐3. The aim of this study was to develop new vaccine candidates against HEV‐3 by investigating alternative vaccine designs using a coordinated approach involving cell culture assays and animal models. We assessed these new candidates in a small animal model (rabbits), characterised resulting antibodies in vitro and evaluated selected candidates in a pig infection model.

In total, seven new vaccine candidates were produced and evaluated using this approach. These candidates included four recombinant protein‐based vaccines and three DNA‐based vaccines. Two protein‐based candidates, p429 and p429‐ORF3, consisted of bacterially expressed central domains of the ORF2 capsid protein, either as a single capsid protein or fused with the ORF3 protein. The ORF3 protein has been proposed as a potentially neutralising target for HEV, as this protein associates with the lipid layer in quasi‐enveloped virions [26], and vaccination with the ORF3 antigen has been shown to induce partial protection against HEV‐1 in rhesus macaques in a previous study [27]. Two further candidates were expressed in insect cells, which consisted of the capsid P‐domain and were either non‐glycosylated (pGS99) or glycosylated (pGS100). A third approach dealt with three DNA‐based vaccines, which consisted of plasmid‐expressed S‐, M‐ and P‐capsid domains, presented either solely or fused with the ORF3 encoding protein at the 5′ or 3′ end. PVax1‐Ub universal fusion vector was used to produce 5′‐ubiquitin‐antigen fusion constructs, which are subjected to enhanced intracellular degradation and improved entry of its epitope peptides into the class I Major histocompatibility complex (MHC) signalling pathway [17, 28]. In addition, these vaccine variants undergo host posttranslational modifications, which can potentially alter the structure and immunological efficacy of the antigen [29]. Our approach could not confirm an enhancement of the immune response, as the humoral response occurred later and antibody levels were lower in the DNA‐based vaccines compared to the protein‐based vaccines. However, Rodriguez et al. found that vaccination with ubiquitin‐coupled DNA vaccines led to a complete abrogation of the humoral immune response in a mouse model [30].

All seven vaccine candidates were administered to individual rabbits and resulting antibodies were examined by testing the corresponding sera. The results showed a faster increase in antibody levels in the four rabbits that were immunised with a protein‐based vaccine, compared to the three rabbits who received a DNA‐based vaccine candidate. Antibodies raised against all vaccine candidates demonstrated binding to HEV‐3 strains in the immunofluorescence staining assay against ‘Kernow C1p6 G1634R’ and ‘pUC83‐2’ (Figure 1C, Figure S4) and exhibited specific, dose dependent neutralising activity against enveloped and naked HEV‐3 in vitro (Figure 1D, Figure S5). Moreover, the binding strength to various epitopes derived from capsid fragments such as pGS99, p239 and p429 was assessed using an avidity ELISA (Figure S6). The resulting data demonstrated that sera from all vaccinated animals exhibited a strong binding affinity, underscoring the efficacy of the vaccines. Based on these tests, vaccines p429 and p429‐ORF3 were identified as the most promising candidates (particularly due to their strong neutralising activity) and subsequently evaluated in a pig infection model. Pigs serve as one of the primary models for HEV infection [31] and are used for evaluating vaccines against HEV‐3 due to their high susceptibility to hepatitis E, with successful infection already upon exposure to low viral doses of 6.5 virus particles [15, 32]. The experiment design including infection procedure and sampling was based on a previously established protocol [15]. Therefore, pigs received two vaccinations with a 4‐week interval and were infected with a HEV‐3 strain 56 days post first vaccination. Both p429 and p429‐ORF3 vaccines elicited a specific antibody response, resulting in significantly lower virus excretion in faeces and delayed onset of viral shedding compared to the non‐vaccinated infection control group (*p* < 0.01 for both vaccines, Figure 2). However, bile samples from a few of the vaccinated pigs tested positive for the virus, indicating that both vaccines offer only partial protection against an HEV‐3 infection and do not confer sterile immunity (Table S1). This is supported by the lack of a consistent increase in serum antibodies or stable antibody titers in either group. Instead, antibody titers declined after the second vaccination and during the infection. This is in contrast to a previous vaccination study with Hecolin and bacterial p239 vaccine in pigs where high antibody titers were maintained until the end of the study on Day 71 [15].

Of particular interest is the first application of ORF2/ORF3 fusion vaccines: The role of ORF3 as a target for neutralising antibodies remains uncertain. Notably, only one out of six piglets in the p429‐ORF3 group developed specific antibodies against ORF3, and this response was also very weak while the immunised rabbit showed a strong antibody response. Additionally, the viral load in the faeces and the virus distribution in the tissues showed no significant difference between the two vaccinated groups. Similarly, no significant differences were observed in viremia across the groups. This might indicate a limited role for ORF3 in the immune response against HEV‐3 in pigs. Therefore, the data imply that the ORF2/ORF3 fusion vaccine might not be as immunogenic in pigs as it is in rabbits, indicating possible species‐dependent differences in immune responses.

Generally, this study focused exclusively on the humoral immune response, which is the major driver of protection by Hecolin as demonstrated in a large clinical Phase III trial [12, 21]. Future examinations of the cellular immune response will provide valuable insights into specific T cell responses triggered by the vaccine. T cells are a critical component of the adaptive immune system and play a central role in recognising and eliminating infected cells.

This study offers a comprehensive framework for selecting new vaccine candidates against HEV‐3, particular for the use in pigs. It includes both extensive in vitro characterisation and thorough in vivo testing in pig models. Vaccinating pigs, the reservoir hosts of HEV, is a promising strategy to prevent transmission within pig populations and especially from pigs to humans. As summarised by Salines et al. (2017), previous studies have reported HEV RNA prevalence rates of 0.8%–10% in pig liver samples, indicating ongoing infection in pigs at the time of slaughter [33]. Similarly, viral RNA was detected in bile samples in this study. Salines et al. identified two key factors associated with a high prevalence of HEV in slaughtered pigs: (1) the presence of intra‐farm circulation of HEV and (2) the timing of infection, with later infections increasing the likelihood that pigs will remain infectious at slaughter [33]. An ideal vaccine would either reduce virus shedding below the infectious threshold to limit herd transmission, or minimise viral loads in edible tissues at slaughter; however, given the high susceptibility of pigs to HEV [2] and the fact that even a relatively low dose of HEV genomes can lead to a substantial risk of infection [3, 32] the ultimate goal is to achieve sterile immunity by completely suppressing viral replication. Therefore, future studies should incorporate additional tissue samples, such as muscle tissue, to ensure HEV clearance in all edible tissues. This targeted approach aligns with the One Health concept and aims to reduce the global burden of hepatitis E.

## Author Contributions

Saskia Weber, Isabella Hrabal, Martin H. Groschup and Martin Eiden designed the research. The experiments were supervised by Martin H. Groschup, Patrick Behrendt and Martin Eiden. Martin Eiden, George Liam Ssebyatika and Thomas Krey designed the vaccine candidates. Isabella Hrabal, Katja Dinkelborg and Elmira Aliabadi conducted the in vitro characterisation of serum samples. Charlotte Schröder provided the animal facilities and animal caretakers. Isabella Hrabal, Saskia Weber, Cora M. Holicki and Laura Schmid performed the in vivo experiment. Christine Fast, Saskia Weber, Cora M. Holicki and Isabella Hrabal conducted the necropsy. Isabella Hrabal and Elmira Aliabadi analysed the animal samples. Isabella Hrabal performed statistical evaluation of results. Isabella Hrabal, Elmira Aliabadi and Martin Eiden wrote the main manuscript text. Isabella Hrabal and Elmira Aliabadi prepared figures and tables. All listed authors reviewed the manuscript.

## Ethics Statement

Rabbit immunisation: notification of the competent authority of the Federal State of Mecklenburg‐Vorpommern, Germany, based on national legislation (LALLF M‐V 7221.3‐2‐042/17). Animal vaccination study in pigs: approval by the State Office for Agriculture, Food Safety and Fishery in the Federal State of Mecklenburg‐Western Pomerania, Germany, based on national and European legislation, EURL 63/2010 for the protection of laboratory animals (LALLF M‐V 7221.3‐1‐010/22).

## Conflicts of Interest

The authors declare no conflicts of interest.

## Supporting information


**Data S1:** liv70246‐sup‐0001‐Supinfo.docx.

**Table S3:** Overview of clinical score (CS) sheets during the pig treatment experiment. See Supporting Information S1 for parameter assignments. If the clinical score differs from 0, the eartag number of the affected.

## Data Availability

All data generated or analysed during this study are included in this published article and its Supporting Information S1 and Table S3.
