# Supplementary material for: Design and Analysis of Novel HEV Vaccine Variants and Evaluation of Two Selected Candidates in a Porcine Infection Model
Source: Liver Int. 2025 Aug 1;45(9):e70246. doi: 10.1111/liv.70246 (PMC12315483; doi:10.1111/liv.70246)
Supplement: Supplementary file 1 — Data S1: liv70246‐sup‐0001‐Supinfo.docx. [file LIV-45-0-s002.docx]

## Supplementary material:

## **Abbreviations**:

*E. coli*  Escherichia coli

ORF open reading frame

RNA ribonucleic acid

ELISA enzyme-linked immunosorbent assay

PBS phosphate-buffered saline

OD optical density

ABTS 2,2'-azino-bis 3-ethylbenzothiazoline-6-sulphonic acid

FCS fetal calf serum

FFU focus forming unit

TBS Tris-buffered saline

TBST Tris-buffered saline with 0.1% Tween

TMB tetramethylbenzidine

AI avidity index

SDS-PAGE sodium dodecyl sulfate-polyacrylamide gel electrophoresis

HRPO horseradish peroxidase

MEM minimal essential medium

NEAA non-essential amino acids

PFA paraformaldehyde

Cy3 cyanine 3

IgG Immunoglobulin G

MFI Mean Fluorescence Intensity

qRT-PCR real-time quantitative reverse transcriptase polymerase chain reaction

ddPCR digital droplet polymerase chain reaction

CS clinical score

## Supplementary sequences

### Supplemental Sequence 1 p429 DNA sequence (1302 nucleotides, 46.9 kDa)

CAT ATG GAG GCG AGC AAC TAT GCG CAA TAT CGG GTT GTT CGC GCA ACC ATC CGC TAT CGT CCT CTG GTT CCG AAT GCG GTC GGC GGC TAT GCG ATC TCC ATT AGC TTT TGG CCG CAG ACG ACT ACA ACC CCT ACG TCG GTC GAT ATG AAC TCT ATC ACC AGC ACA GAC GTA CGC ATT CTC GTA CAA CCG GGT ATT GCC AGC GAA CTG GTC ATT CCC AGC GAA CGT TTA CAC TAC CGC AAC CAA GGC TGG CGT TCC GTG GAG ACT AGT GGC GTA GCT GAG GAA GAA GCC ACG AGT GGG TTG GTC ATG CTG TGC ATC CAT GGG TCA CCA GTC AAC TCG TAC ACG AAT ACG CCG TAT ACT GGC GCT TTA GGC CTC CTT GAC TTT GCG CTG GAG CTC GAA TTT CGC AAT CTT ACG CCA GGC AAT ACC AAT ACC CGC GTT AGT CGC TAC ACC TCG ACA GCA CGT CAT CGT TTA CGC CGT GGT ACC GAT GGA ACC GCG GAA CTG ACT ACC ACA GCC GCA ACT CGG TTC ATG AAG GAT CTG CAC TTT ACC GGG ACC AAT GGC GTG GGC GAA GTT GGG CGC GGT ATT GCG CTG ACG CTG TTC AAT CTG GCG GAT ACG CTG CTT GGT GGC TTA CCG ACG GAA CTG ATT TCC TCT GCC GGT GGT CAG CTC TTC TAC AGC CGC CCT GTG GTA TCC GCC AAC GGA GAA CCC ACC GTG AAA TTG TAC ACG AGC GTG GAG AAT GCC CAG CAA GAT AAA GGG ATT GCG ATC CCG CAT GAC ATC GAT TTG GGT GAC TCA CGC GTT GTG ATT CAG GAT TAT GAC AAC CAG CAT GAA CAG GAT CGC CCG ACC CCG TCA CCA GCC CCG AGT CGT CCG TTT TCG GTG TTA CGC GCG AAT GAT GTC TTG TGG CTG TCA CTG ACC GCA GCT GAA TAC GAC CAG ACT ACC TAT GGC AGC TCT ACA AAC CCG ATG TAC GTT AGC GAT ACC GTG ACT TTC GTG AAC GTA GCC ACA GGT GCC CAA GCT GTT GCG CGT TCG CTG GAT TGG AGC AAA GTG ACG CTG GAT GGA CGT CCA CTG ACC ACC ATT CAG CAG TAT AGT AAG ACC TTC TAT GTG CTG CCG CTG CGC GGA AAA CTG TCC TTT TGG GAA GCG GGT ACT ACG AAA AGT GGC TAT CCC TAC AAC TAC AAC ACA ACG GCA TCT GAC CAG ATT TTG ATC GAG AAC GCC GCA GGC CAC CGT GTG GCA ATC TCT ACC TAT ACC ACC TCG CTG GGT GCA GGC CCT GTG AGC ATT TCC GCG GTT GGT GTC CTT GCT CCG CAT TCA GCT TAA CTC GAG

The sequence was codon optimized for bacterial expression in *E. coli* and synthesized by Eurofins (Eurofins Genomics Europe Shared Services GmbH, Ebersberg, Germany) and cloned via 5’-Ndel and 3’-Xhol restriction sites into the vector pET19b, that harbors a N-terminal His-tag.

### Supplemental Sequence 2 p429-ORF3 DNA sequence (1656 nucleotides, 56.6 kDa)

CAT ATG GAA GCG TCG AAC TAT GCC CAG TAT CGC GTC GTA CGT GCG ACC ATC CGT TAT CGT CCC CTG GTA CCG AAC GCT GTC GGC GGC TAT GCG ATT TCT ATC AGC TTT TGG CCA CAG ACG ACA ACC ACC CCG ACT AGC GTC GAC ATG AAC AGC ATT ACC TCG ACA GAT GTC CGC ATT CTG GTG CAA CCT GGC ATT GCG TCT GAA CTC GTG ATT CCG TCA GAA CGC CTC CAT TAT CGC AAT CAA GGT TGG CGC AGT GTG GAA ACG TCG GGT GTA GCG GAG GAA GAG GCG ACC TCT GGG CTG GTT ATG CTG TGC ATT CAC GGT TCG CCC GTT AAC TCC TAC ACG AAT ACC CCG TAT ACA GGC GCA CTG GGC CTG TTA GAT TTT GCG CTG GAG TTG GAG TTC CGT AAC TTA ACC CCG GGC AAC ACG AAC ACT CGT GTC AGT CGC TAC ACC AGC ACT GCG CGC CAT CGT CTG CGC CGT GGG ACT GAT GGT ACC GCC GAG CTG ACC ACG ACA GCC GCC ACT CGG TTC ATG AAA GAT CTG CAC TTT ACC GGC ACA AAC GGA GTG GGC GAG GTG GGT CGG GGT ATT GCG TTG ACC TTG TTC AAT CTC GCA GAC ACT CTG CTG GGT GGT CTG CCA ACC GAA CTG ATC TCA AGT GCT GGC GGC CAG CTG TTC TAC TCC CGC CCC GTT GTG TCA GCG AAT GGG GAA CCG ACG GTC AAG CTG TAC ACC TCT GTG GAA AAT GCC CAG CAA GAC AAA GGC ATT GCC ATT CCC CAT GAC ATC GAC TTA GGC GAT TCC CGC GTG GTC ATT CAG GAC TAT GAT AAC CAG CAT GAA CAG GAT CGC CCC ACC CCT AGC CCG GCA CCG AGT CGC CCG TTT TCT GTG CTT CGC GCT AAC GAC GTA CTG TGG TTA TCC TTG ACA GCG GCT GAA TAC GAC CAA ACC ACT TAT GGC AGT TCC ACG AAT CCG ATG TAC GTT AGC GAT ACG GTC ACT TTT GTG AAC GTT GCG ACT GGG GCA CAA GCT GTT GCA CGT AGC TTA GAT TGG TCG AAA GTG ACC CTG GAT GGC CGT CCG CTT ACC ACC ATC CAG CAG TAC AGC AAA ACG TTC TAT GTG CTT CCG TTG CGC GGG AAA CTG TCC TTT TGG GAA GCC GGT ACG ACC AAG AGT GGA TAC CCG TAT AAC TAC AAT ACC ACG GCT TCG GAT CAG ATC CTG ATT GAG AAT GCG GCG GGT CAC CGT GTT GCC ATT TCG ACG TAT ACG ACG AGC CTT GGC GCT GGT CCC GTT TCT ATC TCT GCC GTG GGC GTT TTA GCC CCG CAT AGC GCC TTG GCA GAA GCC GCC GCC AAA GAA GCG GCT GCA AAG GAA GCT GCC GCG AAA GAA GCG GCG GCA AAA GCA GCC GCA TGT CCG CGC CAT CGT CCT GCG TCC CGC CTG GCG GTG GTG GTA GGT GGA GCG GCT GCA GTC CCT GCA GTG GTA AGC GGC GTT ACA GGG CTT ATC CCA TCG CCT AGT CCT TCA CCG ATC TTC ATT CAA CCG ACC CCA TTG CCG CCA ACC AGC TTT CAC AAT CCG GGT CTG GAA CTC GCG TTA GAT TCA CGT CCA GCC CCA CTC GTC CCA CTG GGT GCG ACC TCA CCG AGC GCA CCG CCG CTG CCT CCG GTT GTA GAT CTG CCG CAG CTG GGA CTG CGC CGG TAA CTC GAG

The sequence was codon optimized for bacterial expression in *E. coli. It was* synthesized by Eurofins (Eurofins Genomics Europe Shared Services GmbH, Ebersberg, Germany) and cloned via 5’-Ndel and 3’-Xhol restriction sites into the vector pET19b, that harbors a N-terminal His-tag.

The construct encompasses 429 amino acids of open reading frame (ORF) 2, fused to an ORF3 protein, connected via a helix-forming peptide linker (LAEAAAKEAAAKEAAAKEAAAKAA)^1^).

### Supplemental Sequence 3 pVAX1-HEV-SMP DNA sequence (1448 nucleotides, 120.76 kDa)

GGT ACC GGG GCA ATT CTC CGG AGA CAG TAC AAC CTG AGC ACA AGC CCA CTG ACA TCT AGC GTG GCT TCC GGG ACC AAC CTT GTG CTG TAT GCG GCT CCA CTG AAT CCC CTT CTG CCG CTG CAG GAT GGC ACA AAC ACT CAC ATC ATG GCA ACG GAA GCC AGC AAT TAC GCC CAG TAC AGA GTG GTC CGA GCA ACA ATC AGG TAT AGA CCC TTG GTG CCG AAT GCT GTG GGC GGG TAT GCC ATC AGC ATC TCA TTT TGG CCC CAG ACT ACC ACC ACG CCT ACT TCC GTC GAC ATG AAC AGC ATA ACT AGC ACC GAT GTA CGC ATA CTT GTA CAG CCT GGC ATT GCC TCC GAG CTC GTC ATT CCC TCT GAA CGG CTT CAC TAT CGC AAT CAA GGC TGG CGA TCT GTG GAG ACA TCA GGG GTT GCA GAG GAA GAA GCC ACG TCT GGG CTG GTG ATG CTG TGC ATT CAC GGC TCA CCC GTG AAC AGC TAC ACT AAT ACC CCT TAT ACA GGT GCC CTT GGA CTG CTG GAT TTT GCC CTG GAA CTC GAG TTT CGG AAT TTG ACA CCC GGT AAC ACC AAC ACT AGA GTG AGC CGG TAT ACT TCC ACC GCC AGA CAT CGG CTG CGA CGC GGC ACC GAT GGC ACT GCT GAG CTG ACT ACT ACG GCC GCA ACG AGG TTC ATG AAG GAC CTC CAT TTC ACC GGC ACC AAT GGT GTC GGC GAG GTT GGA CGT GGG ATA GCG CTG ACT CTG TTC AAT CTG GCC GAT ACG TTG CTC GGT GGA TTG CCC ACA GAG CTG ATC TCA TCT GCC GGA GGT CAG CTG TTC TAT TCC CGC CCT GTA GTG TCA GCC AAT GGA GAA CCA ACC GTG AAA CTG TAC ACA TCC GTG GAA AAC GCC CAG CAA GAC AAA GGA ATT GCG ATT CCT CAC GAC ATC GAC CTC GGG GAT TCC AGG GTT GTC ATC CAG GAC TAT GAC AAC CAA CAC GAG CAA GAT AGG CCA ACA CCA TCA CCA GCT CCT AGT CGG CCC TTT AGT GTG CTG AGA GCT AAT GAC GTT CTC TGG CTG TCT CTG ACA GCA GCG GAA TAC GAC CAG ACT ACC TAT GGC AGC TCC ACT AAC CCA ATG TAT GTG AGT GAT ACC GTC ACC TTC GTT AAT GTA GCC ACT GGA GCT CAA GCA GTC GCA AGG TCC CTG GAT TGG TCT AAG GTG ACA CTT GAC GGC AGG CCT TTG ACC ACC ATC CAG CAG TAC AGC AAG ACC TTC TAC GTG CTC CCT TTG CGT GGG AAA CTG AGC TTT TGG GAG GCT GGA ACC ACC AAG AGT GGC TAC CCG TAC AAC TAC AAC ACA ACA GCC AGT GAC CAG ATT CTC ATC GAG AAC GCT GCA GGA CAT CGC GTT GCC ATC TCA ACC TAC ACA ACA AGC CTG GGT GCT GGG CCA GTC TCC ATT AGT GCC GTT GGC GTG CTG GCT CCC CAT TCT TGA GCG GCC GC

The sequence was codon optimized for *homo sapiens* as a representant of mammals and synthesized by Eurofins (Eurofins Genomics Europe Shared Services GmbH, Ebersberg, Germany). The sequences were cloned into a modified pVAX1-Ub universal fusion vector that was kindly provided by Friedemann Weber (Institute for Virology, Justus-Liebig-University, Giessen, Germany), which enables expression of a 5'-ubiquitin antigen fusion protein.

### Supplemental Sequence 4 pVAX-HEV-ORF3-SMP DNA sequence (1736 nucleotides, 145.52 kDa)

GGT ACC TGC CCA CGC CAC AGA CCA GCC AGT AGG TTG GCC GTG GTG GTG GGC GGG GCC GCA GCT GTA CCA GCC GTG GTG AGT GGA GTG ACC GGA CTT ATC CCA TCT CCT AGT CCA AGC CCC ATT TTC ATT CAA CCG ACA CCA CTG CCT CCC ACA AGC TTC CAC AAT CCT GGG CTG GAG CTT GCA CTT GAC AGT AGA CCC GCT CCT CTG GTG CCA TTG GGA GCT ACC TCA CCA TCC GCA CCT CCC CTC CCT CCA GTG GTT GAT CTT CCC CAA CTT GGC CTG AGG CGG GGT TCT GGC GGT GCT ATA CTG CGC CGT CAG TAC AAC CTG TCA ACA TCT CCA TTG ACT TCC TCT GTT GCA TCC GGG ACC AAC CTG GTG CTG TAT GCT GCT CCC CTC AAT CCC TTG CTC CCT CTC CAG GAT GGA ACG AAT ACG CAC ATC ATG GCC ACC GAG GCT TCC AAC TAT GCC CAG TAT CGC GTC GTT CGC GCC ACC ATC AGA TAT CGC CCT CTG GTT CCG AAT GCT GTG GGT GGG TAT GCC ATC TCC ATC AGC TTT TGG CCT CAA ACA ACC ACT ACA CCA ACC AGC GTC GAC ATG AAC AGC ATA ACC AGT ACG GAT GTC AGG ATC TTG GTG CAG CCT GGG ATT GCG TCA GAA CTG GTG ATT CCC TCA GAA CGA CTG CAT TAC CGA AAT CAG GGC TGG AGA TCT GTC GAA ACC AGC GGC GTT GCA GAA GAA GAG GCG ACT TCA GGG CTG GTC ATG CTG TGT ATC CAT GGA TCA CCC GTG AAT AGC TAT ACC AAT ACA CCC TAT ACT GGC GCC CTC GGC CTG CTG GAC TTT GCC CTG GAA CTG GAG TTT CGG AAC CTC ACT CCT GGC AAT ACA AAC ACT CGG GTA AGC CGG TAC ACA TCT ACA GCC CGA CAT AGG CTG AGA AGG GGC ACG GAT GGT ACT GCA GAG CTG ACG ACT ACT GCC GCC ACC CGC TTC ATG AAG GAC CTT CAC TTT ACC GGC ACA AAT GGA GTG GGC GAG GTC GGG AGA GGC ATA GCG CTT ACC CTG TTC AAC CTG GCA GAC ACT CTC CTG GGT GGA CTG CCT ACA GAG CTG ATT TCT TCT GCA GGA GGT CAG CTC TTC TAT TCC CGT CCG GTA GTC AGC GCC AAT GGG GAA CCT ACT GTG AAG CTG TAC ACT AGC GTG GAG AAT GCT CAG CAG GAC AAA GGC ATC GCC ATT CCG CAC GAT ATC GAC CTC GGA GAT TCC CGA GTG GTC ATC CAG GAC TAT GAC AAC CAG CAT GAG CAA GAC CGG CCA ACT CCC TCA CCC GCT CCC TCC AGG CCC TTT AGC GTA CTG AGG GCC AAC GAT GTG CTG TGG CTG TCC CTG ACT GCT GCC GAG TAC GAT CAG ACC ACC TAC GGT AGC AGC ACA AAC CCT ATG TAC GTG TCA GAT ACA GTC ACC TTT GTG AAC GTA GCG ACA GGG GCT CAA GCA GTA GCC CGG TCC TTG GAC TGG AGT AAA GTC ACC TTG GAC GGA CGT CCC CTG ACC ACC ATT CAG CAG TAC AGC AAG ACC TTC TAC GTT CTC CCA TTG CGG GGC AAA CTG TCT TTC TGG GAA GCC GGG ACA ACG AAG AGT GGA TAT CCC TAC AAT TAC AAC ACT ACC GCA AGC GAT CAG ATT CTC ATA GAG AAC GCT GCA GGG CAC AGA GTG GCA ATT TCC ACG TAC ACA ACA AGC CTT GGC GCT GGT CCG GTT TCT ATC AGT GCC GTT GGC GTC CTG GCG CCA CAT TCC TGA GCG GCC GC

The sequence was codon optimized for *homo sapiens* as a representant of mammals and synthesized by Eurofins (Eurofins Genomics Europe Shared Services GmbH, Ebersberg, Germany). The sequences were cloned into a modified pVAX1-Ub universal fusion vector that was kindly provided by Friedemann Weber (Institute for Virology, Justus-Liebig-University, Giessen, Germany), which enables expression of a 5'-ubiquitin antigen fusion protein.

### Supplemental Sequence 5 pVAX-HEV-SMP-ORF3 DNA sequence (1736 nucleotides, 145.52 kDa)

GGT ACC GGC GCC ATT CTG CGC AGG CAG TAC AAT CTC TCC ACA TCA CCA TTG ACA TCC TCA GTC GCC TCT GGC ACA AAC CTG GTG CTG TAT GCC GCT CCC CTG AAT CCA CTG CTT CCC CTG CAA GAC GGC ACC AAT ACC CAC ATT ATG GCA ACG GAA GCC TCA AAT TAC GCG CAG TAT CGT GTG GTG AGA GCC ACA ATC CGC TAT CGG CCA CTT GTC CCG AAT GCA GTA GGC GGG TAT GCG ATC TCT ATC AGT TTC TGG CCC CAG ACA ACT ACC ACC CCT ACG AGC GTG GAC ATG AAC AGC ATT ACC TCC ACT GAT GTG CGG ATT CTG GTC CAA CCA GGA ATT GCC AGC GAA TTG GTG ATA CCT AGC GAA AGA CTG CAC TAT CGC AAC CAA GGG TGG CGC TCT GTC GAG ACA TCT GGA GTC GCT GAA GAG GAG GCC ACC AGC GGG CTC GTT ATG CTG TGC ATC CAT GGC TCA CCC GTT AAC AGC TAC ACC AAT ACC CCT TAT ACC GGT GCG CTT GGC TTG CTG GAT TTT GCC CTG GAG CTT GAG TTC CGG AAC TTG ACT CCT GGT AAT ACC AAC ACG AGA GTC TCT CGC TAC ACC AGC ACA GCT CGA CAT AGG CTT CGG AGG GGA ACC GAC GGT ACA GCC GAG CTT ACC ACG ACC GCA GCT ACG CGC TTT ATG AAG GAC TTG CAC TTC ACT GGA ACT AAT GGC GTT GGA GAG GTA GGG AGA GGC ATT GCC CTT ACC CTG TTT AAC CTG GCA GAC ACA CTC CTC GGT GGG CTC CCA ACA GAA CTG ATC TCT TCT GCA GGC GGG CAG CTG TTC TAC AGC AGG CCT GTA GTG AGT GCT AAT GGT GAG CCC ACT GTG AAG CTG TAC ACA TCA GTG GAG AAT GCC CAG CAG GAC AAA GGG ATT GCC ATC CCT CAT GAT ATC GAC CTC GGA GAT TCC CGT GTG GTA ATC CAG GAC TAC GAC AAC CAG CAC GAA CAG GAT AGG CCC ACG CCC AGT CCA GCT CCT AGC AGG CCA TTT TCC GTC CTG AGA GCG AAC GAT GTG CTG TGG TTG TCC CTG ACA GCT GCA GAA TAC GAC CAG ACT ACT TAT GGG TCC AGT ACG AAT CCC ATG TAT GTA AGC GAT ACC GTG ACC TTT GTC AAC GTG GCT ACT GGG GCG CAA GCT GTG GCC CGT TCA TTG GAC TGG TCA AAG GTG ACA CTC GAT GGC CGA CCC CTT ACA ACC ATC CAG CAG TAT TCC AAG ACA TTC TAC GTG CTG CCA TTG CGA GGA AAA CTG TCA TTC TGG GAA GCC GGC ACT ACC AAA TCC GGA TAT CCC TAC AAC TAC AAC ACT ACT GCT TCC GAC CAG ATA CTC ATA GAG AAT GCA GCC GGG CAC AGG GTC GCC ATA AGC ACT TAC ACA ACA AGC CTC GGT GCC GGT CCC GTG TCC ATT AGC GCT GTT GGA GTG CTG GCT CCC CAT AGC GGT AGT GGC TGT CCC AGA CAT CGG CCT GCT TCT CGG CTG GCA GTA GTT GTT GGC GGG GCA GCC GCA GTT CCA GCC GTG GTG AGC GGC GTG ACC GGA CTG ATT CCC TCT CCA AGT CCA AGT CCT ATC TTC ATC CAG CCT ACT CCT CTG CCA CCC ACC TCC TTT CAC AAC CCG GGA CTC GAG CTG GCT CTG GAT AGT CGG CCT GCA CCG CTG GTT CCT CTC GGC GCC ACT AGC CCG TCT GCA CCA CCC CTG CCG CCT GTC GTC GAT CTG CCA CAA CTG GGC CTG CGA AGA TGA GCG GCC GC

The sequence was codon optimized for *homo sapiens* as a representant of mammals and synthesized by (Eurofins Genomics Europe Shared Services GmbH, Ebersberg, Germany). The sequences were cloned into a modified pVAX1-Ub universal fusion vector that was kindly provided by Friedemann Weber (Institute for Virology, Justus-Liebig-University, Giessen), which enables expression of a 5'-ubiquitin antigen fusion protein.

### Supplemental Sequence 6 2xORF3 DNA sequence (648 nucleotides)

Additionally, a separate construct was designed to exclusively express the ORF3 protein as an ORF3-ORF3 fusion protein, with the following sequence:

CAT ATG TGC CCG CGC CAC CGG CCG GCC AGC CGT CTG GCC GTC GTC GTG GGC GGC GCA GCG GCG GTG CCG GCG GTG GTT TCT GGG GTG ACA GGG TTG ATT CCC AGC CCT TCG CCC TCC CCT ATA TTC ATC CAA CCA ACC CCT TTG CCG CCG ACG TCG TTT CAC AAT CCG GGG CTG GAG CTC GCT CTC GAC AGC CGC CCC GCC CCC TTG GTA CCT CTT GGC GCG ACC AGT CCC AGC GCC CCC CCG CTG CCC CCC GTC GTC GAC CTG CCC CAG CTG GGG CTG CGC CGC CTG GCC GAG GCC GCC GCC AAG GAG GCC GCC GCC AAG GAG GCC GCC GCC AAG GAG GCC GCC GCC AAG GCC GCC GCC TGC CCG CGC CAC CGG CCG GCC AGC CGT CTG GCC GTC GTC GTG GGC GGC GCA GCG GCG GTG CCG GCG GTG GTT TCT GGG GTG ACA GGG TTG ATT CCC AGC CCT TCG CCC TCC CCT ATA TTC ATC CAA CCA ACC CCT TTG CCG CCG ACG TCG TTT CAC AAT CCG GGG CTG GAG CTC GCT CTC GAC AGC CGC CCC GCC CCC TTG GTA CCT CTT GGC GCG ACC AGT CCC AGC GCC CCC CCG CTG CCC CCC GTC GTC GAC CTG CCC CAG CTG GGG CTG CGC CGC TAA GGA TCC

The sequence was codon optimized for bacterial expression, synthesized by Eurofins (Eurofins Genomics Europe Shared Services GmbH, Ebersberg, Germany) and cloned into pET19b vector via 5’NdeI and 3’ BamH1 restriction sites. ORF3 proteins were connected via a helix-forming peptide linker (LAEAAAKEAAAKEAAAKEAAAKAA^1^).

Furthermore, the partial HEV capsid protein p239 (nucleotide position 6300-7016 of KP294371) was used as an antigen in ELISA analysis^2^.

## Supplementary Methods

### Supplemental Method 1 Expression and purification of recombinant proteins

Expression of bacterial proteins was performed in *E. coli* BL21(DE3) (Thermo Fisher Scientific, Life Technologies GmbH, Darmstadt, Germany), followed by purification via Ni-NTA columns (Qiagen, Hilden, Germany) under denaturing conditions using standard protocols^3^ (Qiagen manual). The proteins elution fractions were dialyzed against 0.05 M carbonate-bicarbonate buffer pH 10.3 and protein concentration was determined by Nanodrop®(ThermoScientific, Life Technologies GmbH, Darmstadt, Germany) and verified by ROTI®Quant universal (Carl Roth GmbH + Co. KG, Ebersberg, Germany) according to the manufacturer’s instructions and diluted to a final concentration of 500 µg/ml. Proteins were stored at -20°C until use.

For non-secreted and secreted forms of Hepatitis E virus genotype 3 (HEV-3) P domain (pGS99/100, respectively), a stable S2 cell transfectant was established per construct and the proteins were produced as previously described^4^ with minor method modifications. Briefly, a total of 2 µg plasmid (1:1 ratio) was co-transfected with 0.1 µg pCoPuro plasmid^5^. Following a six-day selection period, stable cell lines were then adapted to insect-Xpress media (Lonza, Basel, Switzerland). For large-scale production, *Drosophila S2* cells were induced with 4 µM CdCl2 at a density of 6 x 106 cells/ml. On day 5 post-induction, the non-secreted (pGS99) and secreted (pGS100) P domains were affinity purified from cell lysates and culture supernatants, respectively, using a Strep-Tactin XT 4Flow column (IBA Lifesciences, Göttingen, Germany) followed by size exclusion chromatography on a HiLoad 26/600 superdex 200 pg column (Cytiva Life Sciences, Marlborough, Massachusetts, USA) equilibrated in 20 mM HEPES (Karl Roth GmbH, Karlsruhe, Germany) pH 7.4 and 150 mM NaCl. Purified proteins were concentrated and stored at -80°C until further use.

pVax1 constructs were amplified in *E. coli* K12 DH5α cells (Invitrogen by Thermo Fisher Scientific, Life Technologies GmbH, Darmstadt, Germany) and corresponding plasmids were prepared with the Plasmid Giga Kit (Qiagen, Hilden, Germany) according to the manufacturer’s instructions. Purified plasmids were verified by restriction digest using restriction enzymes HindIII and XhoI (New EnglandBioLabs®inc, Ipswich, Great Britain). Plasmids were diluted to a concentration of 2.5 µg/ml in RNAse free water and finally stored at -20°C until further use. Further details regarding the cloning strategies can be obtained upon request.

### Supplemental Method 2 ELISA (enzyme-linked immunosorbent assay)

An indirect antigen ELISA was performed according to standard protocol^3^ using recombinant antigens HEV p239 , p429 or 2xORF3 respectively. In brief, Nunc MaxiSorp 96 well plates were coated with corresponding antigens at a concentration of 1 µg/ml in 0.05 M carbonate-hydrogen carbonate buffer pH 10.3. Following blocking with 10 % skimmed milk in Phosphate buffered saline (PBS), sera (1:25 diluted in 2% skimmed milk) were added and incubated for 1 hour. After washing with PBS/0.1% Tween, peroxidase conjugated Protein G (EMD Millipore Corp., Darmnstadt, Germany, diluted 1:5000) was applied as the secondary antibody. For visualisation, 2,2'-azino-bis 3-ethylbenzothiazoline-6-sulphonic acid (ABTS) solution was used, and optical density was determined at 405 nm in an ELISA reader (Tecan, Männedorf, Switzerland).

### Supplemental Method 3 Neutralization assay

Rabbit serum samples were heat inactivated (30 minutes, 65°C) and titrated in duplicate in fivefold serial dilutions (starting from a dilution of 1:200) in minimal essential medium (MEM) low IgG fetal calf serum (FCS) medium. Naked and pseudo-enveloped HEV-3 viral strain Kernow C1p6 G1634R were generated following previously described methods^6^. Each serum dilution (40 μl) was mixed with medium (40 μl) containing either the naked or pseudo-enveloped HEV-3 and incubated at 37 °C for 1 hour. Parallel assays were conducted with serum samples from the animals before immunization as negative controls. The resulting serum-virus mixtures were then added to the human hepatoma cell line HepG2/C3A cells seeded in 96-well plates, followed by incubation at 37°C and 5% CO_2_. After 24 hours, 100 µl fresh medium was added. Four days post-infection, cells were fixed with 3% paraformaldehyde (PFA) in PBS and permeabilized with 0.2% Triton. Subsequently, the cells were stained for the ORF2-encoded capsid protein. Focus-forming units (FFUs) were counted using an ELISpot reader (Immunospot, Shaker Hights, Cleveland, UA). The endpoint FFU in the presence of rabbit serum was determined using the formula: 100 × [average FFU in the presence of serum from the immunized animal / average FFU in the presence of serum from the animals before the time point of immunization].

To analyze the neutralization activity of serum samples from the vaccinated pigs against naked HEV, similar experiments were performed using either pig serum samples, diluted in three-fold serial dilutions starting from 1:300, or IgG isolated from pig serum samples, diluted in fivefold serial dilutions starting from 0.16 µg/ml. As negative controls, parallel assays were run with serum samples or isolated IgG from either the control pigs or the vaccinated pigs prior to the time point of vaccination.

### Supplemental Method 4 Urea-based avidity ELISA

To assess the avidity of sera obtained from immunized rabbits and pigs towards various HEV antigens, a urea-based avidity ELISA was established. The procedure involved coating Nunc ImmunIon Maxisorp ELISA (Thermo Fisher Scientific, Life Technologies GmbH, Darmstadt, Germany) plates with recombinant HEV antigens (pGS99, p239 or p429) at a concentration of 1-1.5 µg/ml for 1 hour at 37°C. Subsequently, plates were washed with Tris-buffered saline (TBS) with 0.1% Tween (TBST) and blocked overnight with TBST containing 2.5% non-fat dry milk and 2.5% horse serum at 4°C.

Serially diluted serum samples, starting from 1:20, were added to the plates and incubated at 37°C for 2 hours. Following this incubation, replicate serum samples underwent two 5-minute treatments with 8M urea diluted in TBS solution. After washing, anti-rabbit or anti-pig IgG antibodies were introduced to detect the bound antibodies, and the plates were further incubated at 37°C for 1 hour. Subsequently, Tetramethylbenzidine (TMB) substrate was added to the wells, and plates were incubated for 15 minutes at room temperature. The reaction was stopped by adding 1M Sulphuric acid, and the absorbance was measured at 450 nm with a reference wavelength of 630 nm. The non-linear regression model in GraphPad Prism 10 (GraphPad Prism version 10.0.0 for Windows, GraphPad Software, Boston, Massachusetts USA) was used to analyse the data. The avidity index (AI) was calculated as the serum dilution for half-maximal binding after urea incubation divided by the corresponding value in the absence of urea.

### Supplemental Method 5 SDS-PAGE and Western blot

Sodium dodecyl sulfate-polyacrylamide gel electrophoresis (SDS-PAGE) was carried out with in a 13% bis/acrylamide gel according to standard protocols^3^. Staining was performed using ROTI® Blue quick (Carl Roth, Ebersberg, Germany) following manufacturer’s instructions. For subsequent western blot analysis, proteins were transferred onto Polyvinylidene fluoride (PVDF) membranes by semi-dry electroblotting. The membranes were incubated with blocking buffer (PBS, 0.1% Tween, 5% skim milk powder) at room temperature for 30 minutes, followed by a 60-minutes incubation step with the corresponding primary antibody, diluted 1:50 in 5% skim milk in PBS with 0.1% Tween. After washing in PBS 0.1% Tween, the membrane was incubated with horseradish peroxidase (HRPO) conjugated Protein G (EMD Millipore Corp., Darmstadt, Germany) as a secondary antibody, and subsequently visualized with the chemiluminescent substrate SuperSignal™ West Pico Plus (ThermoScientific, Life Technologies GmbH, Darmstadt, Germany) according to the manufacturer's instructions.

### Supplemental Method 6 Immunofluorescent staining

To investigate the binding of rabbit sera to HEV-3, human hepatoma HepG2/C3A cells were transfected with HEV constructs as described before^6^. The transfected cells were subsequently treated with rabbit sera. Specifically, two distinct plasmids were utilized for transfection: One encoding the full-length HEV-3 “Kernow-C1 p6 clone” and the other the “HEV83-2-27-clone”. Additionally, a plasmid containing the subgenomic Kernow-C1 p6 HEV sequence, coupled to a Gaussia luciferase reporter gene, served as control. The transfection of HepG2 cells was achieved through electroporation, as described before^6^. Staining was performed using an established standard protocol^6^. Five days post-transfection, the cells were fixed with 3% PFA in PBS, followed by permeabilization with 0.2% Triton. Subsequently, the cells were incubated overnight at room temperature with rabbit sera. The next day, the cells were stained using Alexa Fluor 488-conjugated anti-rabbit IgG. (Thermo Fisher Scientific, Life Technologies GmbH, Darmstadt, Germany) Microscopic images were captured at 10x magnification using an Olympus IX2 inverted microscope (Olympus, Tokyo, Japan). The acquired images were then subjected to analysis using ImageJ software^7^ to calculate the Mean Fluorescence Intensity (MFI).

### Supplemental Method 7 IgG isolation

Antibodies from pig serum samples were purified using Melon Gel IgG Spin Purification Kit (Thermo Fisher Scientific, Life Technologies GmbH, Darmstadt, Germany) according to the manufacture’s protocol. Post-purification, the antibody buffer was exchanged with PBS using Amicon ultra centrifugal filters (Merck Millipore, Darmstadt, Germany).

### Supplemental Method 8 Molecular analysis

From the faecal samples, a 25% (w/v) suspension was prepared with 0.89% NaCl solution followed by vortexing and centrifugation at 7,459 x G at 4°C for 10 minutes. 100 µg of tissue samples were homogenized via Tissue lyser II (Qiagen, Hilden, Germany) in 500 µl ZB5 cell culture medium (FLI, Greifswald-Riems, Germany) and also centrifuged. The pellets were discarded.

For extraction, 100 µl of serum, inoculum or fecal/tissue supernatant was used. RNA isolation of serum samples, fecal samples and tissue samples was done with the NucleoVet Mag (Macherey-Nagel, Düren, Germany) kit according to the manufacturer’s instruction using the KingFisher Flex extraction robot (Thermo Scientific, Darmstadt, Germany).

Viral RNA was determined by real-time quantitative reverse transcriptase polymerase chain reaction (qRT-PCR) of the ORF 2/3 overlapping region according to published protocols^8^ using a Bio-Rad CFX96™ Real-Time Systems (Bio-Rad Laboratories, Hercules, California, USA). The amplification of β-actin was used as internal PCR control^8^. For quantification, standards were generated by digital droplet PCR (ddPCR) using the One-Step RT-ddPCR Advanced Kit for Probes 200rxns (Bio-Rad Laboratories, Hercules, California, USA) and the Bio-Rad QX-200® Droplet Reader (Bio-Rad Laboratories, Hercules, California, USA). The HEV-3 RNA for the standards was obtained from the liver tissue of an experimentally infected pig^9^. The samples were classified as HEV-negative if the HEV qRT-PCR results showed a copy number per microliter (copies/µl) below 1 compared to the concurrent standard.

### Supplemental Method 9. Details of the pig vaccination experiment

22 eleven-weeks old male and female mast hybrid pigs were randomly collected from a local commercial pig farm (Landboden Glasin, Glasin, Germany) and tested for the absence of HEV by RT-qPCR and ELISA in blood and fecal samples. All pigs were kept under biosafety level (BSL)-2 conditions in the corresponding animal facilities at the FLI, Germany. Clinical parameters were evaluated daily throughout the experiment using a standardized evaluation form (ScoreSheet). Signs of acute hepatitis or a score of 15 or above on the ScoreSheet were established as termination criteria prior to the start of the trial and would have led to immediate euthanasia of the pigs (Supplemental Data 1).

The group size for was calculated using G*Power 3.1.9.4 ^10^ to generate results with a statistical power of 80% considering a significance level of 5%. The pigs were divided into five experimental groups: Uninfected control group (n=2), infected adjuvant control group (n=2), infection control group (n=6) and two vaccine groups (n=6 each) (Table 2 in the maintext). The pigs were housed in separate stable units, each with 2-3 piglets of the same experimental group and gender. The experimental design is shown in Figure 2A and followed a recently established protocol^2^.

After an acclimation period of seven days, pigs in the vaccine groups received intramuscularly 300 µg (corresponding 600µl protein in carbonate bicarbonate buffer pH 10) of the vaccine, mixed 1:1 (v/v) with Gerbu Adjuvant F (Biotechnik Gerbu, Heidelberg, Germany) into the gluteal muscle group, with a booster on day 29 post vaccination. Adjuvant control pigs received 600 µl of carbonate bicarbonate buffer, mixed 1:1 (v/v) with Gerbu Adjuvant F.

Infection challenge was carried out on day 56 post first vaccination with a 2 ml inoculum (9.5x10^3^ copies/µl) injected intravenously into the cranial vena cava.

Blood and fecal samples were collected every two to three days post infection following a recently established protocol^2^. Four weeks after challenge, the pigs were euthanized by electrical stunning and subsequent exsanguination. Necropsies were performed, and samples from blood, feces, bile, liver, brain, spleen, kidney, cranial mesenteric lymph node and gall bladder wall were stored at −80°C for further analysis.

### Supplemental Method 10 Statistical evaluation

The statistical analysis was performed using R statistical software (R Core Team [2023]. _R: A Language and Environment for Statistical Computing_. R Foundation for Statistical Computing, Vienna, Austria) and GraphPad Prism (GraphPad Prism version 10.0.0 for Windows, GraphPad Software, Boston, Massachusetts USA). Normal distribution was evaluated using the Shapiro-Wilk test, QQ-plot and histogram. Group comparisons were conducted using both the Wilcoxon Rank Sum Test and the Welch two-sample t-test. For the t-test, the null hypothesis (H0) posited that the virus content in samples of vaccinated animals was equal to or greater than that in unvaccinated animals, while the alternative hypothesis (H1) proposed the opposite relationship: H0 = µ(vaccination) ≥ µ(control group); H0 = µ(vaccination) < µ(control group).

## Supplemental Data 1: Evaluation form for clinical parameters of pigs during the pig HEV infection trial

Score sheet for evaluation of pigs during the experiment.

| Date | Animal eartag | Sampling | | Temperature | Liveliness | | | | Posture | | | | Respiration | | | | Movement | | | | Lameness | | | | Skin | | | | Eyes | | | | Feces | | | | Appetite | | | | Feeding behavoiour | | | |
| --- | --- | --- | --- | --- | --- | --- | --- | --- | --- | --- | --- | --- | --- | --- | --- | --- | --- | --- | --- | --- | --- | --- | --- | --- | --- | --- | --- | --- | --- | --- | --- | --- | --- | --- | --- | --- | --- | --- | --- | --- | --- | --- | --- | --- |
|  |  | Feces | Blood |  | 0 | 1 | 2 | 3 | 0 | 1 | 2 | 3 | 0 | 1 | 2 | 3 | 0 | 1 | 2 | 3 | 0 | 1 | 2 | 3 | 0 | 1 | 2 | 3 | 0 | 1 | 2 | 3 | 0 | 1 | 2 | 3 | 0 | 1 | 2 | 3 | 0 | 1 | 2 | 3 |
|  |  |  |  |  |  |  |  |  |  |  |  |  |  |  |  |  |  |  |  |  |  |  |  |  |  |  |  |  |  |  |  |  |  |  |  |  |  |  |  |  |  |  |  |  |
|  |  |  |  |  |  |  |  |  |  |  |  |  |  |  |  |  |  |  |  |  |  |  |  |  |  |  |  |  |  |  |  |  |  |  |  |  |  |  |  |  |  |  |  |  |
|  |  |  |  |  |  |  |  |  |  |  |  |  |  |  |  |  |  |  |  |  |  |  |  |  |  |  |  |  |  |  |  |  |  |  |  |  |  |  |  |  |  |  |  |  |
|  |  |  |  |  |  |  |  |  |  |  |  |  |  |  |  |  |  |  |  |  |  |  |  |  |  |  |  |  |  |  |  |  |  |  |  |  |  |  |  |  |  |  |  |  |
|  |  |  |  |  |  |  |  |  |  |  |  |  |  |  |  |  |  |  |  |  |  |  |  |  |  |  |  |  |  |  |  |  |  |  |  |  |  |  |  |  |  |  |  |  |
|  |  |  |  |  |  |  |  |  |  |  |  |  |  |  |  |  |  |  |  |  |  |  |  |  |  |  |  |  |  |  |  |  |  |  |  |  |  |  |  |  |  |  |  |  |
|  |  |  |  |  |  |  |  |  |  |  |  |  |  |  |  |  |  |  |  |  |  |  |  |  |  |  |  |  |  |  |  |  |  |  |  |  |  |  |  |  |  |  |  |  |
|  |  |  |  |  |  |  |  |  |  |  |  |  |  |  |  |  |  |  |  |  |  |  |  |  |  |  |  |  |  |  |  |  |  |  |  |  |  |  |  |  |  |  |  |  |
|  |  |  |  |  |  |  |  |  |  |  |  |  |  |  |  |  |  |  |  |  |  |  |  |  |  |  |  |  |  |  |  |  |  |  |  |  |  |  |  |  |  |  |  |  |
|  |  |  |  |  |  |  |  |  |  |  |  |  |  |  |  |  |  |  |  |  |  |  |  |  |  |  |  |  |  |  |  |  |  |  |  |  |  |  |  |  |  |  |  |  |
|  |  |  |  |  |  |  |  |  |  |  |  |  |  |  |  |  |  |  |  |  |  |  |  |  |  |  |  |  |  |  |  |  |  |  |  |  |  |  |  |  |  |  |  |  |
|  |  |  |  |  |  |  |  |  |  |  |  |  |  |  |  |  |  |  |  |  |  |  |  |  |  |  |  |  |  |  |  |  |  |  |  |  |  |  |  |  |  |  |  |  |

**Parameter: (Clinical Score = CS)**

Liveliness

- 0 attentive, curious, gets up immediately
- 1 somewhat tired, gets up hesitantly but independently
- 2 listless, only gets up under force, immediately lies down again
- 3 Apathetic, cannot be convinced to get up despite stimulation

Posture

- 0 relaxed posture, straight back
- 1 Stiff posture, arched back
- 2 arched back, stiff movement over a longer period of time
- 3 Severely arched back or does not stand up

feeding behavior

- 0 normal feeding behavior and weight gain, good musculature
- 1 Slightly reduced food intake, normal musculature
- 2 Anorexia, empty belly, weak musculature
- 3 Anorexia, sunken flanks, vertebrae and ribs visible, severe weight loss (over 15% of initial weight)

Respiration

- 0 Physiological, respiratory rate 10-15/min
- 1 Slightly increased respiratory rate, respiratory rate > 20/min
- 2 Moderately increased respiratory rate, respiratory rate > 20/min, clear respiratory movement
- 3 Dyspnea, greatly increased respiratory rate, respiratory rate > 30/min, mouth breathing

Movement

- 0 coordinated movements
- 1 unsteady movement, corrects crossed legs delayed
- 2 Significant ataxia/ weakness of hind legs, able to walk
- 3 Severe signs of paralysis, unable to walk

Lameness

- 0 weight equally distributed on all four legs
- 1 protection of one/several legs, shortened steps
- 2 sparing of one/several legs, weight load on the leg is significantly shortened
- 3 resting of one/several legs, can’t put weight on limbs

Skin

- 0 evenly light pink, bristles not ruffled
- 1 reddened skin areas
- 2 blue-red discolored, cold skin areas, individual punctiform hemorrhages
- 3 black-blue discoloration, no sensitivity, extensive skin bleeding

Eyes/conjunctiva

- 0 pale pink
- 1 reddened, clear secretion
- 2 very red, cloudy secretion
- 3 very red, purulent secretion, vascular injection

Appetite

- 0 greedy for offered food
- 1 eats offered food hesitantly
- 2 does not eat offered food, but sniffs at it
- 3 eats nothing, no interest in food

Defecation

- 0 formed feces, amount physiological
- 1 mild diarrhea
- 2 thin, mushy diarrhea
- 3 watery to bloody diarrhea

Feed in the trough

- (not assessed for the individual animal)
- 0 Trough empty, cleanly eaten
- 1 Trough almost empty, little food left over
- 2 Feed only partially eaten, lots of feed left over
- 3 Trough still full, nothing eaten

The following criteria lead to closer monitoring (at least twice a day):

- Increase in rectal body temperature to over 40°C
- Separation from the group
- Lack of interest in feeding/appetence
- Clinical score > 2 in one or more categories

If a cumulative CS > 5 is reached in an animal or a CS value of 3 is reached in a category, a veterinarian must be consulted immediately and treatment initiated if necessary.

Termination criteria:

Euthanasia under anesthesia is performed immediately if the point total of the CS reaches or exceeds 15 and treatment is not expected to be successful in the opinion of the attending veterinarian or curative treatment that has been initiated has not led to a significant reduction in symptoms.

Animals with a low score but unexpectedly severe clinical symptoms that are not directly assessed with the clinical score are euthanized immediately (e.g. bone fractures, severe joint inflammation).

Definitive general termination criteria:

- Lack of food or fluid intake for more than 24 hours
- Decrease in body weight by > 20% compared to initial weight for more than 2 days
- Animal surface is cold; legs and abdominal skin bluish-dark as a sign of disturbed circulation in combination with shallow, strained breathing
- Severe mutilation (deep skin wounds, bitten toes, etc.)
- Severe central nervous deficits/stuck (e.g. symptoms of high-grade encephalitis such as spastic and central paralysis, clonic convulsions with uncoordinated forced movement and abnormal lying position
- Other symptoms suggesting an unacceptable condition

Specific termination criteria for hepatitis E in pigs

If symptoms of severe acute hepatitis/acute liver failure occur, a veterinarian is called in immediately to assess the clinical condition of the animal and the animal is monitored more closely (at least twice a day). If the symptoms are not reduced by 24 hours or if no significant reduction of symptoms is to be expected in the veterinarian's opinion, the animal has to be euthanized immediately. If more than two of the symptoms described below occur simultaneously, the animal must be removed from the experiment immediately and euthanized.

- Apathy (listlessness)
- Polydipsia (excessive binge drinking)
- Ascites (ascites)
- Icterus (jaundice)
- Vomitus (vomiting)
- Diarrhea
- Coagulation disorder
- Severe cramps
- Hepatoencephalic syndrome/central nervous deficits

## Supplementary Figures

### Supplemental Figure 1


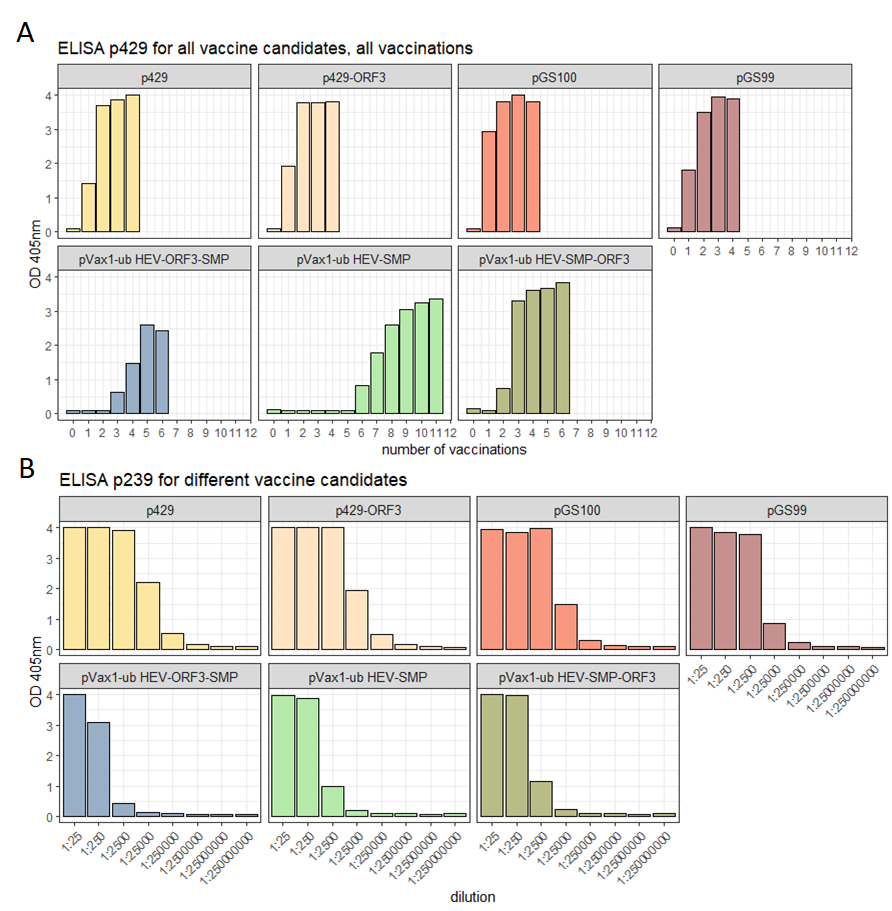


Supplemental Figure 1: (A) p429 ELISA obtained after immunization of rabbits with 0.5 mg/ml protein-based vaccine or 2.5 mg/ml DNA-based vaccine (plasmids). All immunizations included. (B) Serum samples of the rabbits after the last immunization were diluted up to 1:250,000,000 and tested in an ELISA against p239 -coating antigen.

### Supplemental Figure 2


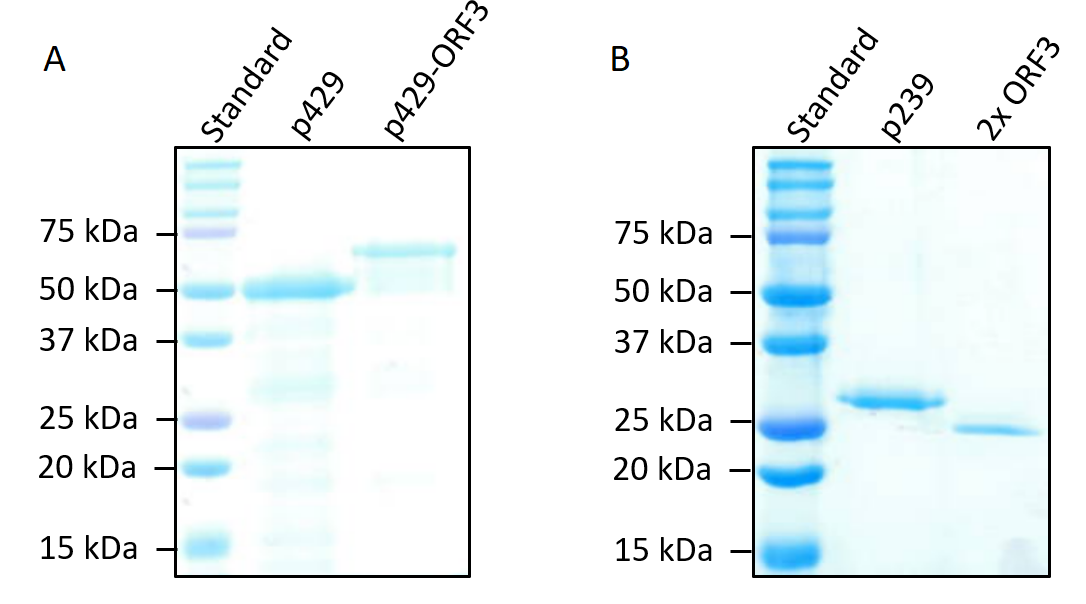


Supplemental Figure 2: (A) SDS-Page analysis and Roti® Blue quick staining of bacterially expressed p429 and p429-ORF3 (left) after dialysis in carbonate bicarbonate buffer pH 10.3. P429 has an expected molecular weight of 46.9 kDa and p429-ORF3 of 58.6 kDa. (B) SDS Page analysis and RotiBlue quick staining of bacterially expressed p239 and 2xORF3. P239 has an expected molecular weight of 25.6 kDa, 2xORF3 of 21.4 kDa after bacterial expression.

### Supplemental Figure 3


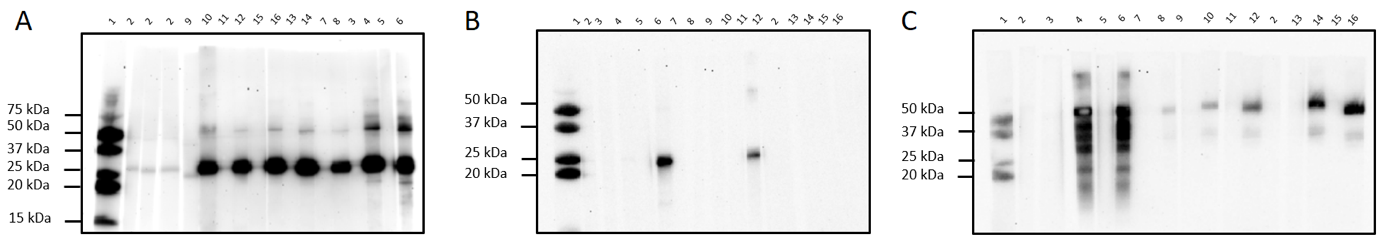


Supplemental Figure 3: Western blot of rabbit sera. Blotted proteins: p239 (A, 25.6 kDa), 2xORF3 (B, 25.6 kDa) and p429 (C, 46.9 kDa). Rabbit serum samples obtained before and immunizations were used as primary antibodies for comparison. Protein G without a primary antibody was used as an additional negative control. The labeling of the serum samples is as follows: (1) Standard, (2) negative control, (3, 4) p429 before and post immunization, (5, 6) p429-ORF3 before and post immunization, (7, 8) pVax1-ub HEV-ORF3-SMP before and post immunization, (9, 10) pVax1-ub HEV-SMP-ORF3 before and post immunization, (11, 12) pVax1-ub HEV-SMP before and post immunization, (13, 14) pGS00 before and post immunization, (15, 16) pGS100 before and post immunization.

### Supplemental Figure 4

**

Supplemental Figure 4: Mean fluorescence intensity (MFI) of the HEV transfected hepatoma (HepG2) cells after incubation with rabbit sera (obtained after the last immunization) as primary antibody and then anti-rabbit IgG as secondary antibody.

### Supplemental Figure 5


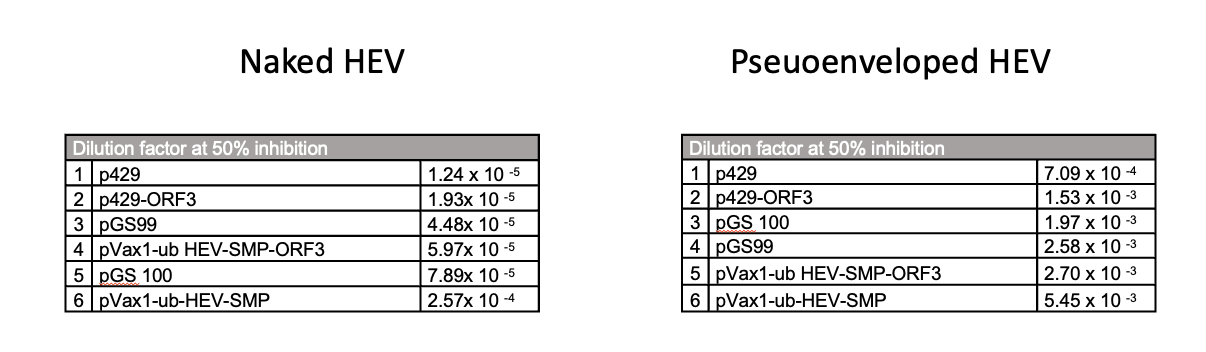


Supplemental Figure 5 Neutralization assay of rabbit sera. The tables present the dilution factors at 50% inhibition for each serum sample from the immunized rabbits against naked and pseudo-enveloped forms of HEV-3 viral strain Kernow C1 p6 G1634R.

### Supplemental Figure 6


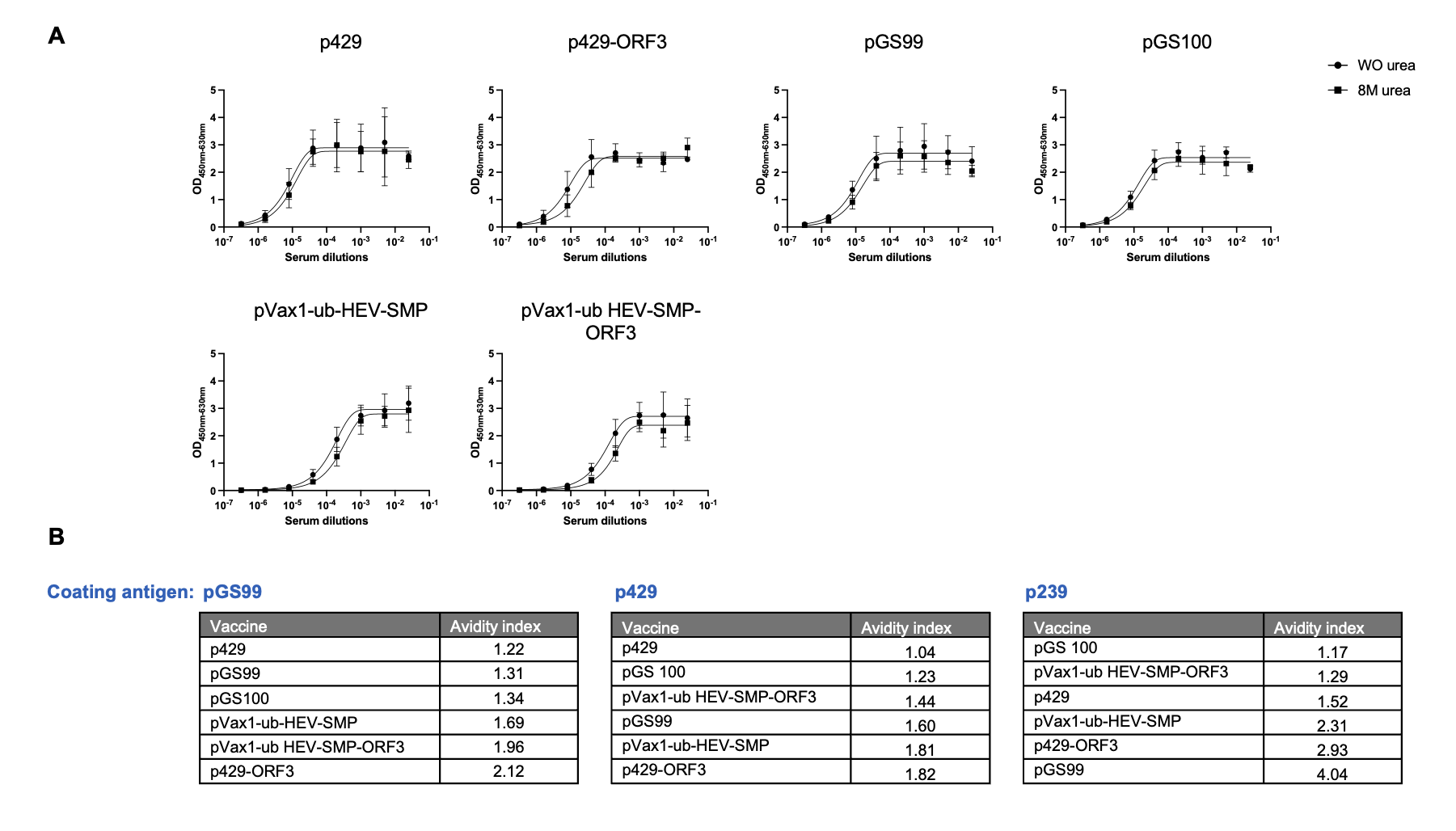


Supplemental Figure 6 Avidity ELISA of rabbit sera. (A) Titration curves of rabbit sera (after the last immunization) using ELISA with pGS99 as the coating antigen, both with and without incubation with 8M urea. Depended on the binding strength, incubation with urea leads to a disruption of the bond and a decrease of absorption. Absorption at 450 - 630 nm is plotted against serum dilution. Sera were titrated in five-fold serial dilutions, starting at 1:40 dilution. (B) The table displays the avidity indices of rabbit sera, calculated as serum dilution for half-maximal binding after urea incubation divided by the corresponding value in the absence of urea.

### Supplemental Figure 7


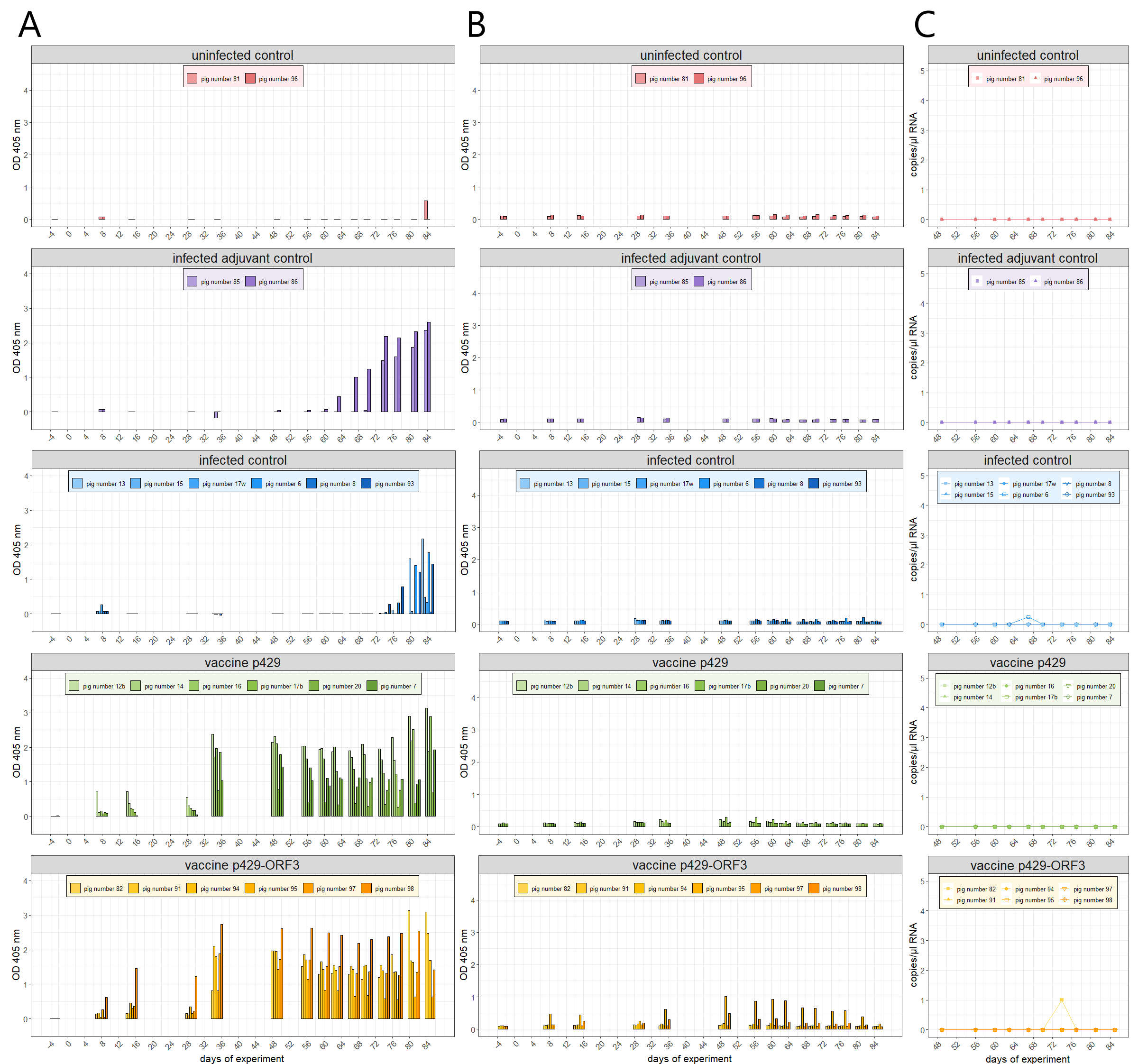


Supplemental Figure 7: Analysis of pig serum samples during the pig infection trial. (A) commercial ELISA (ID Screen ®), (B) ELISA with 2xORF3 coating, (C) detection of viral RNA in serum samples.

### Supplemental Figure 8


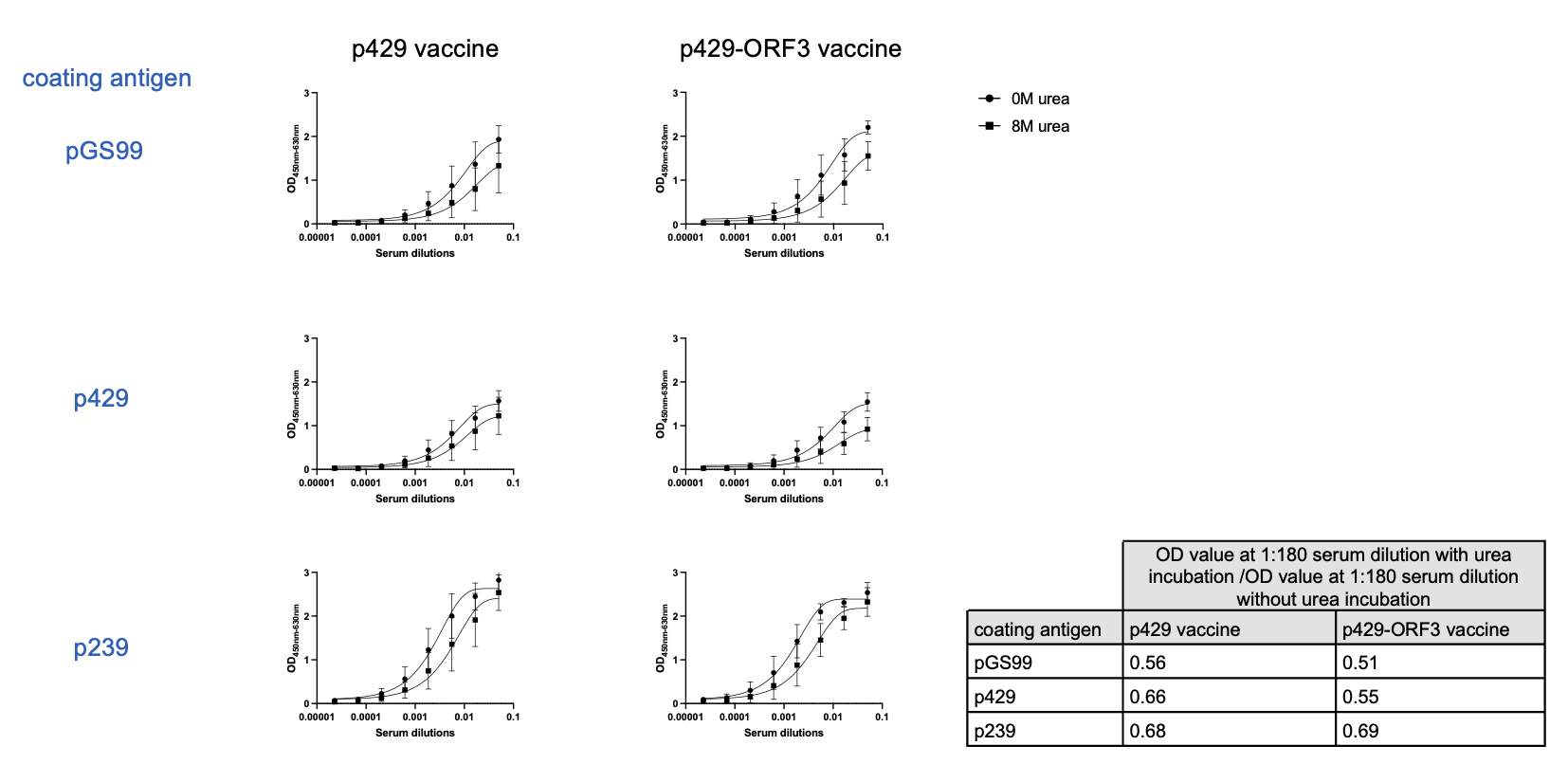


Supplemental Figure 8: Urea-based avidity ELISA from pig serum samples 56 days post first vaccination. The titration curves derived from the mean of ELISA experiments conducted for each vaccine group, employing pGS99, p429 or p239 as coating antigen. Absorption at 450 - 630 nm is plotted against serum dilution. Sera were titrated in five-fold serial dilutions, starting at 1:20 dilution. The table presents the fold change in OD values at the 1:180 serum dilution after urea incubation.

### Supplemental Figure 9


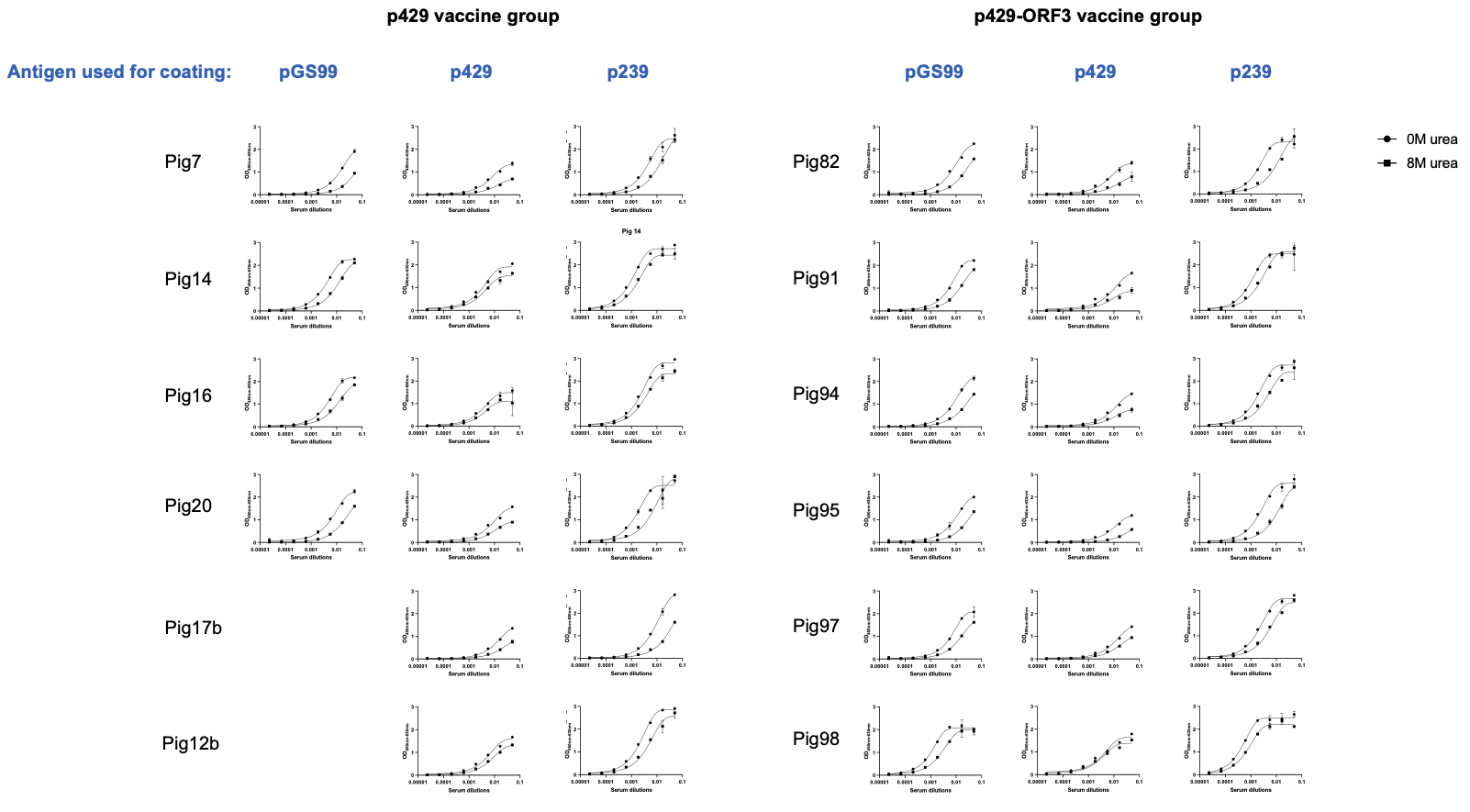


Supplemental Figure 9: Titration curves of pig sera56 days post the first vaccination using ELISA with pGS99, p429 or p239 as the coating antigens, both with and without urea incubation, are presented. Absorption at 450 - 630 nm is plotted against the sera concentration. Sera were titrated in fivefold serial dilutions, starting at a 1:20 dilution.

## Supplementary Tables

### Supplemental Table 1

Supplemental Table 1 Viral RNA in organ samples after necropsy, measured by real time RT-qPCR.

| **Group** | **Animal** | **Tissue (copies/µl RNA)** | | | | | | |
| --- | --- | --- | --- | --- | --- | --- | --- | --- |
|  |  | **brain** | **liver** | **gallbladder** | **spleen** | **kidney** | **Cranial mesenteric lymph node** | **bile** |
| **Uninfected control** | Number 81 | - | - | - | - | - | - | - |
|  | Number 96 | - | - | - | - | - | - | - |
| **Infected adjuvant control** | Number 85 | - | - | - | - | - | - | - |
|  | Number 86 | - | 2.12 | - | - | - | - | - |
| **Infected control** | Number 15 | - | - | - | - | - | - | 2.13 |
|  | Number 17w | - | - | - | - | - | - | - |
|  | Number 93 | - | - | - | - | - | - | - |
|  | Number 6 | - | - | - | - | - | - | 2920.00 |
|  | Number 8 | - | - | - | - | - | - | 70.73 |
|  | Number 13 | - | - | - | - | - | - | - |
| **Vaccine p429** | Number 12b | - | - | - | - | - | - | - |
|  | Number 14 | - | - | - | - | - | - | - |
|  | Number 20 | - | - | - | - | - | - | - |
|  | Number 7 | - | - | - | - | - | - | - |
|  | Number 16 | - | - | - | - | - | - | - |
|  | Number 17b | - | - | - | - | - | - | 43.83 |
| **Vaccine p429-ORF3** | Number 82 | - | - | - | - | - | - | - |
|  | Number 94 | - | - | - | - | - | - | 67.76 |
|  | Number 97 | - | - | - | - | - | - | - |
|  | Number 91 | - | - | - | - | - | - | 1605.00 |
|  | Number 95 | - | - | - | - | - | - | 193.60 |
|  | Number 98 | - | - | - | - | - | - | - |

### Supplemental Table 2

Supplemental Table 2: Overview of p values obtained by statistical testing. Data are positive skewed distributed. The statistical analysis of the animal test data was performed using the R statistical software (R Core Team [2023]. _R: A Language and Environment for Statistical Computing_. R Foundation for Statistical Computing, Vienna, Austria). In the context of the one-tailed t-test, the null hypothesis (H0) proposed that the mean of the vaccinated treatment group exceeded that of the infected control group, while the alternative hypothesis (H1) suggested the contrary: H0 = µtreatment ≥ µinfected control; H1= µtreatment < µinfected control. p-values < 0.05 are marked in green, p-values < 0.1 are marked in yellow.

|  | Welch-two-sample-t-test (one-tailed) | Welch-two-sample-t-test (two-tailed) | Wilcoxon Rank Sum Test |
| --- | --- | --- | --- |
| HEV-RNA in serum (infected control group vs. P429-Vaccine group) | p-value = N/A | p-value = N/A | W = 4095, p-value = 0.3173 |
| HEV-RNA in feces (infected control group vs. P429-Vaccine group) | p-value = 0.0001614 | p-value = 8.119e-05 | W = 13222, p-value = 1.447e-07 |
| HEV-RNA in liver (infected control group vs. P429-Vaccine group) | p-value = NA | p-value = NA | W = 18, p-value = NA |
| HEV-RNA in bile (infected control group vs. P429-Vaccine group) | p-value = 0.3568 | p-value = 0.1784 | W = 24.5, p-value = 0.2155 |
| HEV-RNA in serum (infected control group vs. P429-Vaccine group, exclusion of pigs without HEV-3 RNA) | p-value = N/A | p-value = N/A | W = 2730, p-value = 0.4142 |
| HEV-RNA in feces (infected control group vs. P429-Vaccine group, exclusion of pigs without HEV-3 RNA) | p-value = 0.002422 | p-value = 0.001211 | W = 8498, p-value = 0.00038 |
| HEV-RNA in liver (infected control group vs. P429-Vaccine group, exclusion of pigs without HEV-3 RNA) | p-value = NA | p-value = NA | W = 12, p-value = NA |
| HEV-RNA in bile (infected control group vs. P429-Vaccine group, exclusion of pigs without HEV-3 RNA) | p-value = 0.3601 | p-value = 0.1801 | W = 15.5, p-value = 0.4005 |
| HEV-RNA in serum (infected control group vs. P429-ORF3-Vaccine group) | p-value = 0.32 | p-value = 0.84 | W = 4050.5, p-value = 0.9937 |
| HEV-RNA in feces (infected control group vs. P429-ORF3-Vaccine group) | p-value = 0.0001562 | p-value = 7.809e-05 | W = 13276, p-value = 2.54e-07 |
| HEV-RNA in liver (infected control group vs. P429-ORF3-Vaccine group) | p-value = NA | p-value = NA | W = 18, p-value = NA |
| HEV-RNA in bile (infected control group vs. P429-ORF3-Vaccine group) | p-value = 0.7421 | p-value = 0.371 | W = 17.5, p-value = 0.9319 |
| HEV-RNA in serum (infected control group vs. P429-ORF3-Vaccine group, exclusion of pigs without HEV-3 RNA) | p-value = 0.3206 | p-value = 0.8397 | W = 3368, p-value = 0.9038 |
| HEV-RNA in feces (infected control group vs. P429-ORF3-Vaccine group, exclusion of pigs without HEV-3 RNA) | p-value = 0.00172 | p-value = 0.0008598 | W = 10864, p-value = 1.277e-05 |
| HEV-RNA in liver (infected control group vs. P429-ORF3-Vaccine group, exclusion of pigs without HEV-3 RNA) | p-value = NA | p-value = NA | W = 15, p-value = NA |
| HEV-RNA in bile (infected control group vs. P429-ORF3-Vaccine group, exclusion of pigs without HEV-3 RNA) | p-value = 0.8325 | p-value = 0.4163 | W = 13, p-value = 0.7017 |
| HEV-RNA in serum (infected adjuvant control group vs. P429-Vaccine group) | p-value = NA | p-value = NA | W = 1350, p-value = NA |
| HEV-RNA in feces (infected adjuvant control group vs. P429-Vaccine group) | p-value = 0.09195 | p-value = 0.04597 | W = 3800.5, p-value = 0.07493 |
| HEV-RNA in liver (infected adjuvant control group vs. P429-Vaccine group) | p-value = 0.5 | p-value = 0.25 | W = 9, p-value = 0.08326 |
| HEV-RNA in bile (infected adjuvant control group vs. P429-Vaccine group) | p-value = 0.3618 | p-value = 0.8191 | W = 5, p-value = 0.5637 |
| HEV-RNA in serum (infected adjuvant control group vs. P429-Vaccine group, exclusion of pigs without HEV-3 RNA) | p-value = NA | p-value = NA | W = 900, p-value = NA |
| HEV-RNA in feces (infected adjuvant control group vs. P429-Vaccine group, exclusion of pigs without HEV-3 RNA) | p-value = 0.3508 | p-value = 0.1754 | W = 2414, p-value = 0.5057 |
| HEV-RNA in liver (infected adjuvant control group vs. P429-Vaccine group, exclusion of pigs without HEV-3 RNA) | p-value = NA | p-value = NA | W = 6, p-value = 0.1573 |
| HEV-RNA in bile (infected adjuvant control group vs. P429-Vaccine group, exclusion of pigs without HEV-3 RNA) | p-value = 0.391 | p-value = 0.8045 | W = 3, p-value = 0.4795 |
| HEV-RNA in serum (infected adjuvant control group vs. P429-ORF3-Vaccine group) | p-value = 0.32 | p-value = 0.84 | W = 1335, p-value = 0.5637 |
| HEV-RNA in feces (infected adjuvant control group vs. P429-ORF3-Vaccine group) | p-value = 0.08484 | p-value = 0.04242 | W = 3809, p-value = 0.0984 |
| HEV-RNA in liver (infected adjuvant control group vs. P429-ORF3-Vaccine group) | p-value = 0.5 | p-value = 0.25 | W = 9, p-value = 0.08326 |
| HEV-RNA in bile (infected adjuvant control group vs. P429-ORF3-Vaccine group) | p-value = 0.2861 | p-value = 0.857 | W = 3, p-value = 0.2519 |
| HEV-RNA in serum (infected adjuvant control group vs. P429-ORF3-Vaccine group, exclusion of pigs without HEV-3 RNA) | p-value = 0.3206 | p-value = 0.8397 | W = 1110, p-value = 0.5271 |
| HEV-RNA in feces (infected adjuvant control group vs. P429-ORF3-Vaccine group, exclusion of pigs without HEV-3 RNA) | p-value = 0.2085 | p-value = 0.1042 | W = 3101, p-value = 0.2514 |
| HEV-RNA in liver (infected adjuvant control group vs. P429-ORF3-Vaccine group, exclusion of pigs without HEV-3 RNA) | p-value = 0.5 | p-value = 0.25 | W = 7.5, p-value = 0.1138 |
| HEV-RNA in bile (infected adjuvant control group vs. P429-ORF3-Vaccine group, exclusion of pigs without HEV-3 RNA) | p-value = 0.2949 | p-value = 0.8526 | W = 2, p-value = 0.1998 |

References

1. Arai R, Ueda H, Kitayama A, Kamiya N, Nagamune T. Design of the linkers which effectively separate domains of a bifunctional fusion protein. *Protein Eng*. 2001;8:529-532.

2. Dähnert L, Aliabadi E, Fast C, et al. Immunisation of pigs with recombinant HEV vaccines does not protect from infection with HEV genotype 3. *One Health*. 2024:100674.

3. Jäckel S, Eiden M, Balkema-Buschmann A, et al. A novel indirect ELISA based on glycoprotein Gn for the detection of IgG antibodies against Rift Valley fever virus in small ruminants. *Research in Veterinary Science*. 2013;2:725-730.

4. Backovic M, Krey T. Stable Drosophila Cell Lines: An Alternative Approach to Exogenous Protein Expression. In: Murhammer DW, ed. *Baculovirus and Insect Cell Expression Protocols*. Springer New York; 2016:349-358.

5. Iwaki T, Figuera M, Ploplis VA, Castellino FJ. Rapid selection of Drosophila S2 cells with the puromycin resistance gene. *BioTechniques*. 2003;3:482-486.

6. Todt D, Friesland M, Moeller N, et al. Robust hepatitis E virus infection and transcriptional response in human hepatocytes. *Proc Natl Acad Sci U S A*. 2020;3:1731-1741.

7. Schneider CA, Rasband WS, Eliceiri KW. NIH Image to ImageJ: 25 years of image analysis. *Nat Methods*. 2012;7:671-675.

8. Vina-Rodriguez A, Schlosser J, Becher D, Kaden V, Groschup M, Eiden M. Hepatitis E Virus Genotype 3 Diversity: Phylogenetic Analysis and Presence of Subtype 3b in Wild Boar in Europe. *Viruses*. 2015;5:2704-2726.

9. Schlosser J, Eiden M, Vina-Rodriguez A, et al. Natural and experimental hepatitis E virus genotype 3 - infection in European wild boar is transmissible to domestic pigs. *Vet Res*. 2014;1.

10. Faul F, Erdfelder E, Lang A-G, Buchner A. G*Power 3: a flexible statistical power analysis program for the social, behavioral, and biomedical sciences. *Behav Res Methods*. 2007;2:175-191.
